# Media and strain studies for the scaled production of *cis*-enone resorcylic acid lactones as feedstocks for semisynthesis

**DOI:** 10.1038/s41429-021-00432-3

**Published:** 2021-06-21

**Authors:** Zeinab Y. Al Subeh, Huzefa A. Raja, Jennifer C. Obike, Cedric J. Pearce, Mitchell P. Croatt, Nicholas H. Oberlies

**Affiliations:** 1 Department of Chemistry and Biochemistry, The University of North Carolina at Greensboro, Greensboro, NC USA; 2 Mycosynthetix, Inc., Hillsborough, NC USA

**Keywords:** Structure elucidation, Fungal biology

## Abstract

Resorcylic acid lactones (RALs) with a *cis*-enone moiety, represented by hypothemycin (**1**) and (5*Z*)-7-oxozeaenol (**2**), are fungal secondary metabolites with irreversible inhibitory activity against protein kinases, with particularly selective activity for inhibition of TAK1 (transforming growth factor beta-activated kinase 1). Gram-scale quantities of these compounds were needed as feedstock for semi-synthesizing RAL-analogues in a step-economical fashion. To do so, this study had three primary goals: identifying fungi that biosynthesized **1** and **2**, enhancing their production by optimizing the fermentation conditions on the lab scale, and developing straight forward purification processes. After evaluating 536 fungal extracts via an in-house dereplication protocol, three strains were identified as producing *cis*-enone RALs (i.e., MSX78495, MSX63935, MSX45109). Screening these fungal strains on three grain-based media revealed enhanced production of **1** by strain MSX78495 on oatmeal medium, while rice medium increased the biosynthesis of **2** by strain MSX63935. Furthermore, the purification processes were improved, moving away from HPLC purification to utilizing two to four cycles of resuspension and centrifugation in small volumes of organic solvents, generating gram-scale quantities of these metabolites readily. In addition, studying the chemistry profiles of strains MSX78495 and MSX63935 resulted in the isolation of ten other RALs (**3**-**12**), two radicinin analogues (**13**-**14**), and six benzopyranones (**15**-**20**), with **19** and **20** being newly described chlorinated benzopyranones.

## Introduction

The fungal kingdom, which is estimated to include between 2.2 and 5.1 million species represents a large reservoir for a variety of bioactive compounds [1, 2]. It is well known that growth conditions and fermentation media have a significant impact on the fungal biosynthetic machinery [3]. Both the yield and the composition of fungal secondary metabolites are affected by environmental factors [4–10]. The “One-Strain-Many Compounds” approach (OSMAC) was popularized by Bode et al. [11] as a powerful strategy to increase the number of secondary metabolites available from one microbial source, and this involves the alteration of easily accessible cultivation parameters, i.e., media composition. For example, we have recently used the OSMAC approach to produce verticillins as a feedstock for the generation of semisynthetic analogues [6, 12].

Resorcylic acid lactones (RALs) are a class of fungal macrolactone polyketides that have received significant attention due to a wide range of pharmacological properties, including antibiotic, antifungal, antimalarial, antiparasitic, antiviral, anabolic, cytotoxic, estrogenic, immunosuppressive, nematocidal, and sedative activities [13–16]. More specifically, interest in RALs has increased since the discovery of their potent inhibitory activity against several oncogenic protein kinases [17]. In particular, *cis*-enone containing RALs, represented by hypothemycin (**1**) and (5*Z*)-7-oxozeaenol (**2**), act as irreversible inhibitors of a select few protein kinases by forming stable Michael addition products with cysteine residues in the ATP-binding pocket [18, 19]. Given the vital role of protein kinases in the development, progression, and aggressiveness of cancer [20], these natural products have promise as anticancer drug leads. Accordingly, we and others [21–25] have probed analogues of hypothemycin (**1**) and (5*Z*)-7-oxozeaenol (**2**) for their kinase inhibitory activities.

A goal of the current study was to leverage our experience in natural products chemistry with expertise in synthetic chemistry [21, 26–28]. Building from our knowledge of mycology and fungal metabolites, we strove to develop methods to generate the RALs on a scale that facilitates synthetic chemistry efforts. We have previously characterized a suite of RALs from various fungal species, such as *Halenospora* sp., *Phoma* sp., and *Setophoma* sp. [29–31]. For example, (5*Z*)-7-oxozeaenol and its derivatives were isolated from a *Phoma* sp. (now identified as *Setophoma* sp., strain MSX63935) and evaluated for effects on cancer cells [30, 32, 33]. Using those RALs as starting materials, seven semisynthetic analogues of (5*Z*)-7-oxozeaenol were synthetized and investigated for transforming growth factor beta-activated kinase 1 (TAK1)-inhibitory activities, where the novel nonaromatic difluoro-derivative of (5*Z*)-7-oxozeaenol inhibited TAK1 at the nanomolar level [21].

The development of *cis*-enone RAL analogues that retain TAK1 inhibitory activity can be achieved through two main pathways: total synthesis from simple starting materials or semisynthesis *via* altering the compounds isolated from nature. While total synthesis of a variety of RALs has been disclosed [14], total synthesis of *cis*-enone RALs is often challenged by the high number of the reactions involved, the low overall yields, and the need to install the *cis*-enone at a late stage to avoid isomerization to the more stable trans-isomer [34, 35], which lacks activity against TAK1 [21]. Comparatively, it is attractive to use the naturally occurring *cis*-enone RALs as starting points to perform chemical modifications. This semisynthetic approach could facilitate the generation of a large number of analogues in very few synthetic operations, i.e., a more step-economical approach [36–40]. Semisynthesis is not without its own set of challenges, and the limited supply of the naturally occurring RALs is paramount, as evidenced by the high cost of the two main *cis*-enone RALs (e.g., $569 for 1 mg of **1** and $185 for 1 mg of **2** from Sigma-Aldrich) [41]. Thus, the generation of gram-scale quantities of **1** and **2** was approached *via* three interrelated goals. First, fungi known to biosynthesize *cis*-enone RALs were identified. Next, the fermentation conditions were probed, with the goal of developing cost-efficient methods that could be implemented on the gram-scale. Finally, the techniques to purify **1** and **2** from fungal extracts were optimized. In addition, a benefit of scaling up the fermentation procedures was the identification of new compounds that are minor constituents.

## Results and discussion

### Identifying fungal strains that biosynthesize *cis*-enone RALs

**Fig. 1:**
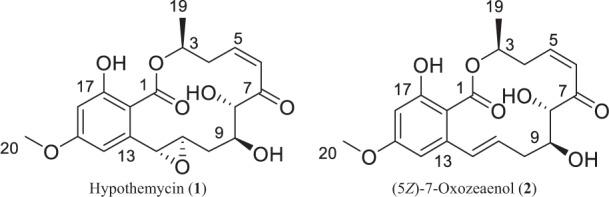
Structures of hypothemycin (**1**) and (5*Z*)-7-oxozeaenol (**2**)

Over the past decade, our research team has built an in-house library of secondary metabolites isolated from filamentous fungi [42, 43]. This database, which includes over 625 compounds, is used to probe the metabolite profile of fungal extracts, and to date, at least 536 fungal cultures have been screened with our dereplication protocol. Among these, seven fungal strains were identified as biosynthesizing RALs (data not shown), and three of those produced *cis*-enone RALs (i.e., hypothemycin (**1**) and (5*Z*)-7-oxozeaenol (**2**)) (Fig. 1 and S1). Accordingly, fungal strains MSX78495, MSX63935, and MSX45109 were chosen to investigate their potential for yielding gram-scale amounts of **1** and **2**. Interestingly, while two of the strains (i.e., MSX78495 and MSX63935) produced both compounds, strain MSX45109 seemed to only generate **1**, albeit at a very low level (Fig. S1).

### Media studies to enhance the production of *cis*-enone RALs

To enhance the production of hypothemycin (**1**) and (5*Z*)-7-oxozeaenol (**2**) on the gram- scale, solid-state fermentation cultures of the three *cis*-enone RAL-producing fungi were examined on rice, oatmeal, and Cheerios (Figs. S2 and S3), due to previous experience with these media as a starting point for enhanced biosynthesis of fungal metabolites [4, 6]; each of those conditions were studied as biological triplicates. While we have employed scores of other media, both defined and rich, we find that these three are a cost effective way to begin the process, turning to other media only if necessary [9]. Subsequently, the cultures were extracted and subjected to UPLC-HRESIMS to confirm the production of the targeted compounds (i.e., **1** and/or **2**), to measure their relative abundance among the various growth conditions, and to determine the preferred fungal strain and culture medium for the highest yields (Figs. S4 and S5).

**Fig. 2:**
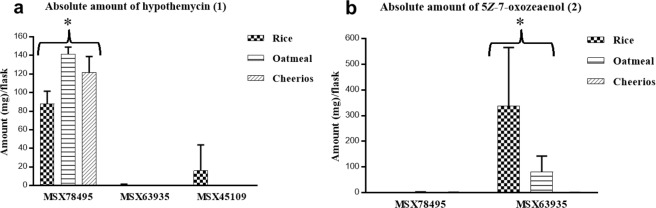
**a**, **b** show the absolute amounts of hypothemycin (**1**) and (5*Z*)-7-oxozeaenol (**2**), respectively, across cultures grown on rice, oatmeal, and Cheerios media. An asterisk Indicates significantly higher productions of hypothemycin (**1**) by strain MSX78495 in panel (a) or (5*Z*)-7-oxozeaenol (**2**) by strain MSX63935 in panel (b), compared to other fungal strains (*p* < 0.05) as demonstrated in Tables S1 and S2. Data are presented as mean ± SD. One of the three biological replicates of strain MSX63935 on rice medium did not show optimal growth, which caused the large error bars in (**b**) for the absolute amount of **2** from strain MSX63935 grown on rice

Based on the UPLC-HRESIMS chromatograms (Fig. S5), hypothemycin (**1**) was detected in the extracts of strains MSX78495, MSX63935, and MSX45109, while (5*Z*)-7-oxozeaenol (**2**) was detected only in the former two strains (Fig. 2). Since the growth medium is known to affect the secondary metabolite profile of fungal cultures [3], the relative abundance of both **1** and **2** were measured across the three strains and three different culture media (Fig. S6). For **1**, strain MSX78495 showed the highest production, with oatmeal delivering the most robust results, relative to rice and Cheerios (Fig. S6). Interestingly, while **1** could be detected under at least one growth condition for all three strains (Fig. S5), the biosynthesis by strain MSX78495 was far superior Fig. S6). On the other hand, the highest biosynthesis of **2** was observed with fungal strain MSX63935 fermented on rice medium (Fig. S6). In contrast to each other, strain MSX78495 biosynthesized **1** on all three media, with a preference for oatmeal, whereas with strain MSX63935, the production of **2** was by far the best on rice medium, with nearly zero production when fermenting with Cheerios. Studies are warranted to investigate how the biosynthesis of two structurally-related compounds could be so different. During our study, one of the three biological replicates of strain MSX63935 grown on rice medium did not show optimal growth as compared to the other two cultures. This caused the manifestation of large error bars in Figs. S6b and 2b for the relative and absolute amount of **2** from strain MSX63935 (Fig. S4). Thus, this culture was excluded from all further isolation and purification processes discussed below.

The absolute amounts of **1** and **2** among the various extracts were measured by developing calibration curves for these two RALs (Fig. S7 and Table S3). Accordingly, the amount of **1** produced by the fungal strain MSX78495 was 87.9 ± 13.6 mg/flask, 141.3 ± 7.8 mg/flask, and 121.8 ± 17.0 mg/flask using rice (10 g/flask), oatmeal (10 g/flask), and Cheerios (7 g/flask) media, respectively (Fig. 2a). Strain MSX78495 was a superb producer of **1**, with a slight, although not statistically significant, preference for oatmeal media. Growing fungal strain MSX63935 on rice allowed for the production of 337.9 ± 227.9 mg per flask of (5*Z*)-7-oxozeaenol (**2**) as compared to 80.0 ± 61.8 mg per flask of oatmeal medium (Fig. 2b). While the error bars for these latter measurements were somewhat large (Fig. 2b), the general trend was approximately a fourfold improvement in biosynthesis of **2** when fermenting strain MSX63935 on rice vs. oatmeal.

### Improved procedures for the isolation and purification of hypothemycin

**Fig. 3:**
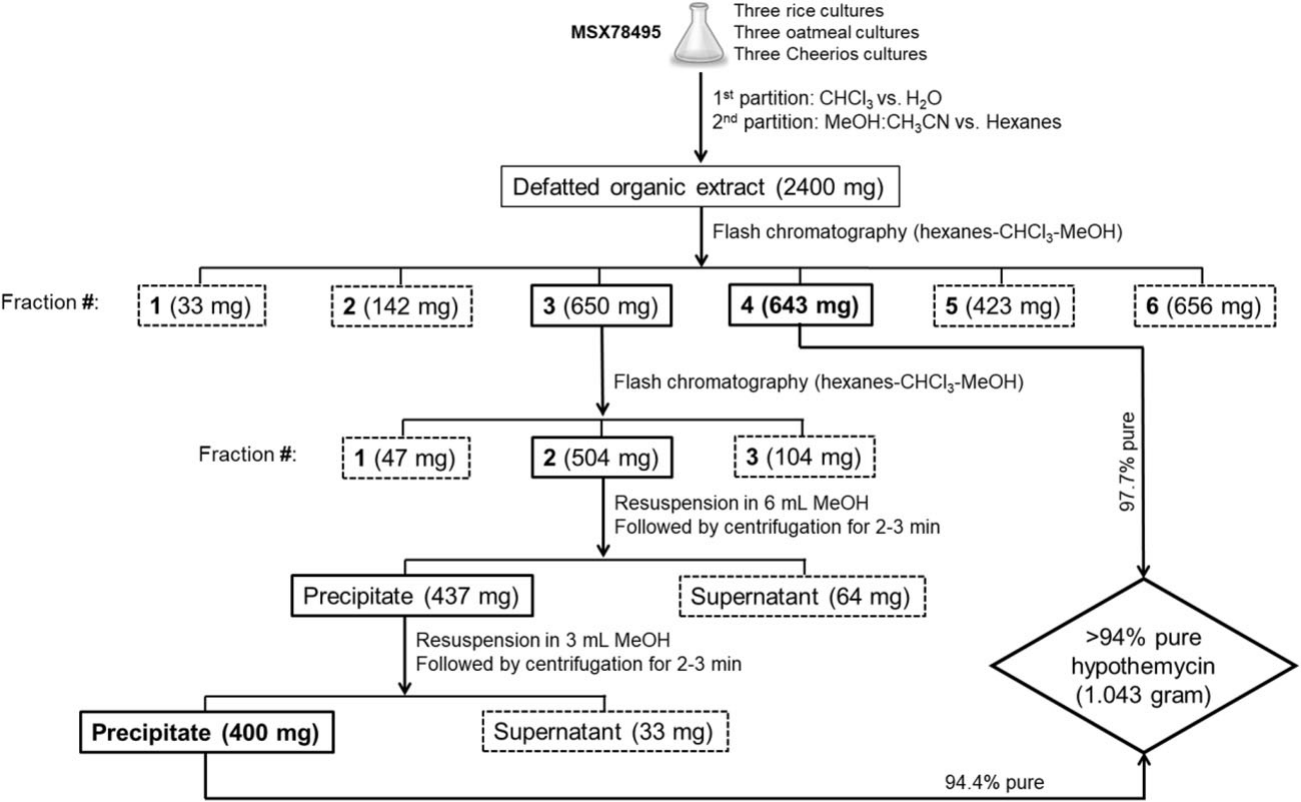
Illustration of the isolation and purification protocol of hypothemycin (**1**), showing how **1** can be generated both from a column fraction and via centrifugation. Over a gram of **1** was generated from nine flasks: three fermented on rice, three fermented on oatmeal, and three fermented on Cheerios. We predict that the yield would be even higher by ~20% if all nine flasks were grown on oatmeal

With enhanced fermentation procedures in hand via strain MSX78495, we sought to next optimize the isolation and purification of **1**. To do so, the extracts obtained from strain MSX78495 grown on the three different culture media were combined and subjected to a first round of fractionation via normal phase flash chromatography to obtain six fractions (Fig. 3 and S8). The fourth fraction (643 mg) was found to be over 97% pure hypothemycin (**1**) as noted by ^1^H NMR and UPLC-PDA data (Fig. S9). In addition, the adjacent fraction (i.e., fraction 3; 650 mg) was ~68% **1** as indicated by analogous ^1^H NMR and UPLC-PDA data (Fig. S10). To further purify fraction 3, a second round of flash chromatography was performed using nearly identical procedures (data not shown) to obtain a fraction (~500 mg) that was enriched in **1**(~89% pure). This sample was subjected to two cycles of resuspension in small volumes of HPLC-grade MeOH (6 ml for the 1st cycle and 3 ml for the 2nd cycle) followed each time by centrifugation for 5 min at 14000 RPM (g force of ~15340) to yield a pelleted sample of **1** (>94% pure; 400 mg; Fig. S11). The supernatants from the 1st and the 2nd cycle of centrifugations were collected (64 mg and 33 mg, respectively), and their ^1^H NMR spectra were compared to that of the precipitate (Fig. S12). While hypothemycin (**1**) was a major component in the supernatant samples, most of the impurities and/or other secondary metabolites were efficiently removed into the supernatant, leaving a >94% pure precipitate of **1**. Following this simplified purification procedure, more than 1 g of hypothemycin (**1**) was obtained rapidly, without the use of HPLC, and at a level of purity that was suitable feedstock for semi-synthetic chemistry efforts. Moreover, the amount of **1** “lost” in the supernatant was relatively low as compared to the total recovered amount of pure hypothemycin (i.e., ~60 mg of **1** recovered from supernatant samples vs. 1040 mg of **1** obtained in the precipitate).

### Improved isolation and purification of (5*Z*)-7-oxozeaenol

**Fig. 4:**
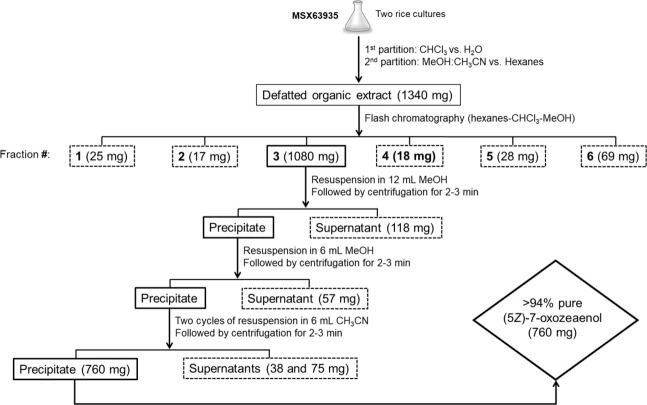
Illustration of the isolation and purification protocol of (5*Z*)-7-oxozeaenol (**2**), showing how **2** can be generated both from a column fraction and via centrifugation. As 760 mg of **2** were generated from two flasks fermented on rice, we predict that over a gram of **2** could be generated with this procedure from three flasks of strain MSX63935 grown on rice

Extracts from the fungal strain MSX63935 grown on rice showed the highest abundance of (5*Z*)-7-oxozeaenol (**2**) (Fig. 2) and therefore were used for the isolation studies. By investigating the three biological replicates grown on rice, the second culture did not grow well as evidenced by a lower amount of extract, and thus less of **2**, as compared to the other two cultures (Fig. S4). While this resulted in a large error bar, we felt it was important to be transparent about this fact. Accordingly, the spurious extract was excluded from the purification process. The other two extracts were combined and subjected to flash chromatography to obtain 1080 mg of the third fraction (Fig. 4 and S13), which was enriched for (5*Z*)-7-oxozeaenol (**2**) as noted in the ^1^H NMR spectrum and UPLC-PDA chromatogram (Fig. S14; ~68% **2**). Further purification of this fraction was performed by following the resuspension/centrifugation technique described above for **1** with some modifications. In this case, instead of pursuing another round of flash chromatography, two cycles of resuspension and centrifugation in HPLC-grade MeOH (12 ml for the 1st cycle and 6 ml for the 2nd cycle) were followed by another two cycles of resuspension and centrifugation in HPLC-grade CH_3_CN (6 ml per cycle); each time, the centrifuge time and force was the same as noted for **1**. In each case, the precipitates were collected, with a final yield of 760 mg of **2** (>94% pure; Fig. S15). The supernatants from the four cycles of centrifugations were collected (i.e., 118 mg, 57 mg, 39 mg, and 75 mg, respectively), and their ^1^H NMR spectra were compared to that of the precipitate (Fig. S16). As expected, and despite the presence of **2** as a main constituent in the supernatant samples, the amount of other secondary metabolites was high. Similar to our experience with **1**, the streamlined procedure, which did not require HPLC, was used to generate more than 750 mg of **2**, which are now being used as feedstock in semi-synthetic chemistry studies, similar to those reported previously [21]. Again, the amount of **2** that was “lost” in the supernatant samples was relatively low (i.e., ~138 mg) and can be recovered by 1–2 injections via prep-HPLC, as described below.

### Chemical profile of hypothemycin-producing fungal strain (MSX78495)

**Fig. 5:**
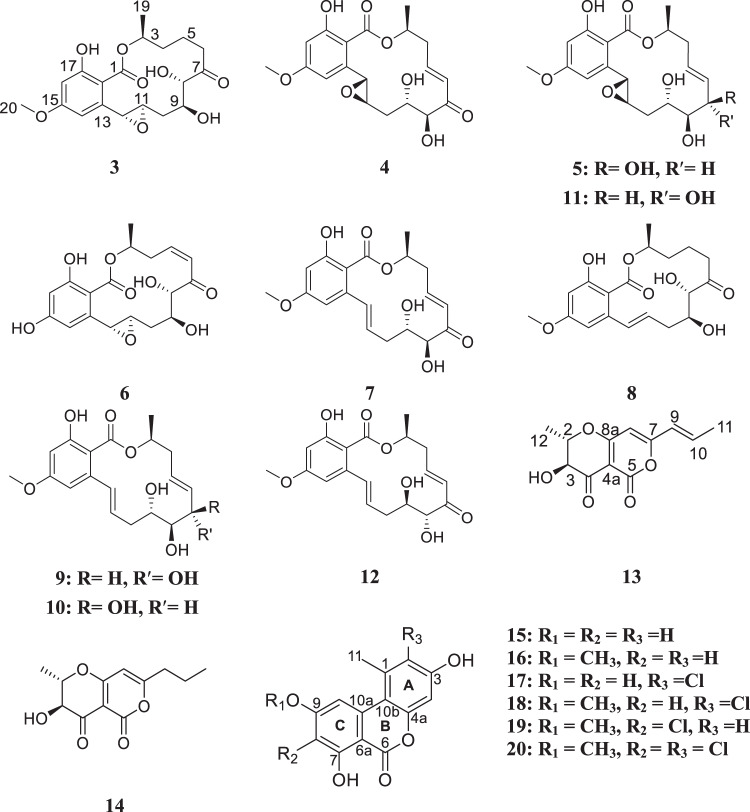
Fungal secondary metabolites produced by strains MSX78495 and MSX63935

Purification of hypothemycin (**1**) from cultures of strain MSX78495 required its separation from other metabolites produced by this fungus. To study the other minor constituents, the supernatants were subjected to preparative high-performance liquid chromatography (HPLC) to obtain six RALs. In addition to **1** [44] and **2** [30, 45], dihydrohypothemycin (**3**) [46], aigialomycin A (**4**) [46], paecilomycin A (**5**) [47], and 4-*O*-demethylhypothemycin (**6**) [48] were isolated (Fig. 5), and their NMR data compared favourably to literature values (see Figs. S17–S22 for ^1^H and ^13^C NMR spectra of **1**-**6**).

### Chemical profile of (5*Z*)-7-oxozeaenol-producing fungal strain (MSX63935)

The fungal strain MSX63935 exhibited more chemical diversity and higher sensitivity to culture medium as compared to strain MSX78495 (Fig. S5). Investigating the content of the collected supernatant via preparative HPLC showed that strain MSX63935 was producing three classes of fungal secondary metabolites. In addition to the RALs (**1**-**2** and **7**-**12**), two radicinin analogues (**13**-**14**) and six benzopyranones (**15**-**20**) were isolated (Fig. 5). ^1^H and ^13^C NMR data were used for identifying these compounds as hypothemycin (**1**), (5*Z*)-7-oxozeaenol (**2**), (5*E*)-7-oxozeaenol (**7**) [30], LL-Z1640-1 (**8**) [49], zeaenol (**9**) [50], 7-*epi*-zeaenol (**10**), aigialomycin B (**11**) [46], cochliomycin F (**12**) [51], radicinin (**13**) [52], dihydroradicinin (**14**) [53], alternariol (**15**) [54], alternariol 9-methyl ether (**16**) [55], rhizopycnin D (**17**) [56], palmariol B (**18**) [55], and the two new benzopyranones (**19-20**). ^1^H and ^13^C NMR spectra of **7**-**18** are presented in Figs. S23-S34.

Compound **19** was obtained as a white amorphous powder with HRESIMS data matching that observed for palmariol A and B (**18**) [55, 57]. The isotopic pattern in the mass spectrum indicated the presence of one chlorine atom in **19**, i.e., molecular ion peaks [M + H]^+^ of *m/z* 307 and 309 in a 3 to 1 ratio (Fig. S35). The NMR data indicated the presence of twelve aromatic carbons, one carbonyl, an aromatic methyl, a methoxy group, and three aromatic protons (Table 1 and Fig. S36). Two of the aromatic protons (i.e., H-2 and H-4) showed meta-coupling with a *J* value of 2.3 Hz indicating their presence on a tetrasubstituted aromatic ring (Table 1 and Fig. S38). The HMBC correlations of H-2 with C-3, C-4, C-10b, and C-11 and H-4 with C-2, C-3, C-4a, and C-10b confirmed the structure of ring A (Figs. S39 and S46). The third singlet aromatic signal at *δ*_H_ 7.23 (H-10) indicated the presence of a pentasubstituted aromatic ring. The HMBC correlations of H-10 with C-10a and C-10b established the connections between ring A and C through ring B. The HMBC correlations of the methoxy protons with C-9 and C-10 confirmed its position at C-9 (Figs. S39 and S46). The position of the chlorine atom at C-8 was suggested by the HMBC correlation of H-10 with C-8, in addition to the chemical shift of C-8 (*δ*_C_ 105.8).

**Table 1:** ^1^H and ^13^C NMR spectroscopic data for **19** and **20** in DMSO-*d*_*6*_

	19^a^		20^b^	
Position	*δ*_C_, type	*δ*_H_ (*J*, Hz)	*δ*_C_, type	*δ*_H_ (*J*, Hz)
1	138.3, C		135.5, C	
2	118.0, CH	6.71, d (2.3)	120.3, C	
3	158.9, C		155.2, C	
4	101.6, CH	6.59, d (2.3)	101.9, CH	6.89, s
4a	152.5, C		150.2, C	
6	164.4, C		164.2, C	
6a	99.5, C		100.3, C	
7	158.3, C		158.1, C	
8	105.8, C		106.4, C	
9	160.7, C		160.7, C	
10	99.7, CH	7.23, s	101.2, CH	7.38, s
10a	136.1, C		135.5, C	
10b	108.4, C		110.1, C	
11	24.9, CH_3_	2.73, s	20.9, CH_3_	2.92, s
CH_3_O-9	56.5, CH_3_	4.01, s	56.9, CH_3_	4.09, s

^a^Recorded at 400 MHz for ^1^H and 100 MHz for ^13^C

^b^Recorded at 500 MHz for ^1^H and 125 MHz for ^13^C

In a similar fashion, the molecular formula of **20** was deduced by HRESIMS data as C_15_H_10_Cl_2_O_5_ (Fig. S41). The molecular ion peaks [M + H]^+^ of m/z 341, 343, and 345 at a 9:6:1 ratio confirmed the presence of two chlorine atoms in **20**. The NMR data indicated the presence of two singlet aromatic protons, instead of the three observed in **19** (Table 1 and Fig. S42). The absence of meta-coupling between these two aromatic protons suggested the position of the second chlorine atom at either C-2 or C-4, and the HMBC correlation of H_3_-11 with C-2 confirmed the position of the second chlorine atom at C-2 (Figs. S44 and S46).

The relatively small molecular weight of these two benzopyranones (**19** and **20**), their low hydrogen to carbon ratio, and the sparsity of ^1^H-^1^H coupling imparts difficulty in confirming the positions of the aromatic substituents. Therefore, NOESY experiments were used to further facilitate the structure elucidation of these compounds, and this approach has been implemented for other hydrogen-deficient natural products [10]. The NOESY correlations between H-2/CH_3_-11, CH_3_-11/H-10, H-10/CH_3_O-9 confirmed the structure of **19** (Figs. S40 and S47). Similarly, compound **20** showed NOESY correlations between CH_3_-11/H-10, H-10/CH_3_O-9, CH_3_O-9/CH_3_-11 suggesting the replacement of the aromatic proton at C-2 with a second chlorine atom (Figs. S45 and S47). The structures of **19** and **20** were similar to palmariol A and B, which are mono-chlorinated benzopyranones isolated from *Lachnum palmae* and *Hyalodendriella* sp. [55, 57]. Palmariol A has a chlorine attached to C-4, while palmariol B has a chlorine attached to C-2. Thus, compounds **19** and **20** were ascribed the trivial names palmariol C and palmariol D, respectively.

## Discussion and conclusion

Herein, the OSMAC approach was used to enhance the biosynthesis of *cis*-enone RALs from three different fungal strains. Interestingly, the effect of fermentation media, i.e., rice, oatmeal, and Cheerios, on the production of these compounds was different across the three fungal strains. Hypothemycin (**1**) was detected in the extracts of the three fungal strains (MSX78495, MSX63935, and MSX45109), which agreed with the previously reported isolation of **1** from MSX63935 and MSX45109 [30, 31]. However, the yield of **1** was significantly higher in fungal strain MSX78495 as compared to the latter two strains (Fig. 2 and S6), and while growth media did not have a large impact on the chemical profile, oatmeal medium allowed for the highest yield of **1**. Despite the structural similarity between (5*Z*)-7-oxozeaenol (**2**) and **1** (i.e., its epoxide derivative), fungal strain MSX63935 was the main producer of **2** as compared to strain MSX78495 (Fig. 2 and S6). Moreover, culture media had a significant impact on the biosynthesis of **2**, where rice medium allowed for a higher yield as compared to oatmeal, while production was nearly eliminated in Cheerios. The effect of fermentation medium on the production of **1** from *Aigialus parvus* was reported previously, where culture medium and its initial pH were the most important factors that affect the production of **1** [58]. However, that study was for growth in liquid media, which yielded 13.6 mg per g of biomass. In addition, no studies have been reported that optimized the production of **2** on the lab scale or compared the production of **1** vs **2**.

The isolation of **1** and **2** and their separation from other secondary metabolites in the culture extracts was another aspect addressed in scaling-up the biosynthesis of these compounds. Our usual isolation protocol involves the fractionation of the defatted culture extract *via* flash chromatography to obtain three to five fractions, followed by one to three rounds of purification using reverse-phase HPLC [29, 30]. As is well known to this audience, preparative HPLC has become the main isolation tool of natural products in the last several decades [59], and this obviously works quite well for milligram quantities of secondary metabolites. However, its application in gram-scale isolation can be challenging and resource intensive, and with **1** and **2**, scaling their purification *via* HPLC is limited by the low solubility of these compounds. For example, HPLC can be used to isolate **1** and **2**, but the co-elution of dihydrohypothemycin (**3**) with **1** and LL-Z1640-1 (**8**) with **2** became problematic when injecting a 50–100 mg sample (Figs. S48 and S49). Accordingly, using a reverse-phase HPLC system for the purification of gram quantities of **1** and **2** would require 10 to 15 repeated cycles of prep-HPLC, which would consume large quantities of organic solvents and produce larger amounts of mixed solvent waste. This is in addition to the long extract-to-purification period, personnel and instrumentation time, and the associated high cost of this procedure (Table S4). A simplified purification process was achieved by optimizing the separation method at an early stage via flash chromatography and applying the concept of resuspension and precipitate collection from small solvent volumes. The facts that **1** and **2** were the main constituents in the original extracts, and that both exhibit relatively low solubility in solvents like MeOH and CH_3_CN, allowed for the enrichment of these two compounds by sedimentation in a sub-gram to gram quantity (Figs. 3 and 4). As evidenced by the composition of the secondary metabolites isolated from the collected supernatants (Fig. 5), two to four cycles of resuspension/precipitate collection were efficient for purifying the targeted two *cis*-enone RALs from a mixture that includes compounds from the same structural class and/or other different classes. Table S4 compares the application of reverse-phase HPLC vs. the resuspension/centrifugation technique in the purification of **1** and **2**. Moreover, the ultimate goal is to use **1** and **2** as feedstock for semi-synthetic efforts, as will be reported in the future, and thus, having a larger quantity (i.e., gram-scale) was more important than the final purity, since synthesis products will be purified in the final step.

In conclusion, *cis*-enone RALs are fungal metabolites with promising activities that could be explored as anticancer drug leads. Scaling up the production of these compounds from the milligram to gram scale required the identification of the best producing fungal strain, modifying fungal growth conditions to enhance their biosynthesis, and the implementation of practical and time efficient purification techniques. Applying the above three-pronged approach allowed for the isolation of 1043 mg of hypothemycin (**1**), 760 mg of (5*Z*)-7-oxozeaenol (**2**), and the identification of 18 (**1**-**18**) known and two new (**19**-**20**) secondary metabolites.

## Materials and methods

### General experimental procedures

Ultraviolet (UV) spectra were measured using a Varian Cary 100 Bio UV‒Vis spectrophotometer (Varian Inc.). 1D and 2D NMR data were obtained using a JEOL ECA-500 spectrometer operating at 500 MHz or a JEOL ECS-400 spectrometer operating at 400 MHz that is equipped with a high sensitivity JEOL Royal probe and a 24-slot autosampler (both from JEOL Ltd.). Residual solvent signals were utilized for referencing (for CDCl_3_ *δ*_H_/*δ*_C_ 7.26/77.16 and for DMSO-*d*_6_ *δ*_H_/*δ*_C_ 2.50/39.52). UPLC-HRESIMS data were collected via an LTQ-Orbitrap XL mass spectrometer (Thermo Finnigan, San Jose, CA, USA) equipped with an electrospray ionization source (ESI) and connected to a Waters Acquity UPLC system. A BEH Shield RP18 column (Waters, 1.7 µm; 50 × 2.1 mm) was used and heated to 40 °C. The flow rate of the mobile phase was 0.3 ml min^−1^ and consisted of a gradient system of 15:85 to 100:0 of CH_3_CN-H_2_O (0.1% formic acid) over 10 min. MS data were collected from *m/z* 150 to 2000 in the positive mode. A Varian Prostar HPLC system, equipped with ProStar 210 pumps and a Prostar 335 photodiode array detector (PDA), was used to conduct all analytical and preparative HPLC experiments, with data collected and analyzed using Galaxie Chromatography Workstation software (version 1.9.3.2, Varian Inc.). Flash chromatography was performed on a Teledyne ISCO CombiFlash Rf 200 using Silica Gold columns (from Teledyne Isco) and monitored by UV and evaporative light‒scattering detectors. An Eppendorf 5415 centrifuge, equipped with rotor F-45-18-11, was used for the resuspension/centrifugation of fractions enriched in hypothemycin (**1**) and (5*Z*)-7-oxozeaenol (**2**).

### Fungal strains identification

Mycosynthetix fungal strain MSX45109 was isolated from leaf litter collected in a mangrove habitat in 1989 [31]; MSX63935 was isolated in 1992 from leaf litter collected at an agricultural farm [30]; and MSX78495 was collected on terrestrial leaf litter in 1993 in a semi humid gallery forest. These three strains were isolated by Dr. Barry Katz. Molecular techniques were used to identify the three strains by sequencing the internal transcribed spacer regions 1 & 2 and 5.8 S nrDNA (ITS) [60, 61] with primers ITS1F and ITS4 [62, 63]. As MSX45109 was identified in a previous study as *Setophoma terrestris* [31], we hypothesized that the two other RAL producing strains may have phylogenetic affinities to *Setophoma*. A BLAST search using the RefSeq Database in NCBI GenBank showed that strains MSX63935 and MSX78495 had ≥90% sequence homology with members of *Setophoma* spp. [64]. Hence, we downloaded all previously described species of *Setophoma* from recently published literature [65–69] and constructed a multiple sequence alignment in MUSCLE using the program Seaview [70]. The alignment was trimmed to remove ambiguous characters using GBlocks [71]. ModelFinder was used to select the best-fit model using Akaike Information Criterion [72]. The best fitting substitution model: transversion model with empirical base frequencies, allowing for a proportion of invariable sites, and a discrete Gamma model with four rate categories (TVM + F + I + G4) was determined by AIC. The trimmed alignment was then used to infer the Maximum Likelihood of ITS sequence data using IQ-TREE implemented in PhyloSuite [73]. Ultrafast bootstrapping was done with 5000 replicates [74]. Nodes with UFBoot ≥90% are shown on the clades but only nodes ≥95% were considered strongly supported. Based on results of the Maximum Likelihood analysis using IQ-Tree, the MSX strains showed phylogenetic affinities with *Setophoma* and were nested within the *Setophoma* clade (Fig. S50), but we could not assign a species name to strains MSX63935 and MSX78495. It is likely these two strains represent putative new isolates; however additional phenotypic data along with multigene data from LSU, *tub2*, *tef-1α* and *gapdh* (i.e., a polyphasic approach) needs to be undertaken to determine their exact identities. Herein, we identify the two strains as *Setophoma* spp. (*Phaeosphaeriaceae, Pleosporales Ascomycota*). The ITS sequences of the two new strains were deposited in the GenBank (accession numbers: MSX63935: MW881143, MW881144; MSX78495: MW881145, MW881146).

### Media and fermentations

The cultures of fungal strains MSX78495, MSX63935, and MSX45109 were maintained on potato dextrose agar (PDA; Difco) and were transferred periodically to fresh PDA plates. An agar plug from the leading edge of the PDA culture was transferred to a sterile tube with 10 ml of YESD (2% soy peptone, 2% dextrose, and 1% yeast extract). The YESD culture was grown for 7 days on an orbital shaker (100 rpm) at room temperature (~23 °C) and then used to inoculate three types of solid fermentation media.

As previously described [4], cultures of each fungal strain were grown in three different grain-based media in triplicate: rice, breakfast oatmeal, and Cheerios breakfast cereal [75] for a total of 27 cultures (Fig. S2 and S3). Solid-state fermentations were carried out in 250-ml Erlenmeyer flasks. To prepare rice medium, 10 g of rice were added to each flask with 20 ml of deionized water (DI H_2_O). For the oatmeal medium, the same amount was used in each flask with 17 ml of DI H_2_O. For Cheerios medium, 7 g of Cheerios were used in each flask without water. After autoclaving these samples at 120 °C for 20 min, the flasks were inoculated with YESD seed cultures (described above) and incubated at room temperature for 2 weeks. Over the incubation period, the fungal cultures grew normally with no sign of growth retardation, except for the MSX63935 cultures growing on Cheerios medium, which were dried out by the time of extraction (Fig. S3).

### Extraction, fractionation, and isolation

The extraction procedure was described previously [4]. Briefly, each flask of solid culture was extracted with 90 ml of 1:2 CH_3_OH-CHCl_3_ and vacuum filtered. To the filtrate, 90 ml of CHCl_3_ and 100 ml of DI H_2_O were added, and the mixture was stirred for 30 min and then transferred into a separatory funnel. The bottom layer was drawn off and evaporated to dryness. The dried organic extract was re-constituted in 100 ml of 1:1 CH_3_OH-CH_3_CN and 100 ml of hexanes. The CH_3_OH/CH_3_CN layer was drawn off and evaporated to dryness under vacuum. The extract amounts that were produced by each culture are shown in Fig. S4. Before performing further fractionation, quantitative UPLC-HRESIMS data were collected. After that, the extracts of MSX78495 grown on various media were combined to give a total of 2.4 g of extract material, while the extracts of MSX63935 grown on rice were combined to provide 1.34 g of extract material. Both extracts from the fungal strains MSX78495 and MSX63935 were subjected to normal-phase flash chromatography to obtain six fractions each using a gradient solvent system of hexanes-CHCl_3_-CH_3_OH at a 35 ml min^−1^ flow rate. The hypothemycin-containing fraction (from MSX78495) and the (5*Z*)-7-oxozeaenol-containing fraction (from MSX63935) were identified *via* UPLC-HRESIMS. Further purification of **1** and **2** was achieved by following the outlined work-flows (Figs. 3 and 4, respectively). The supernatant collected from the resuspension/centrifugation process of the hypothemycin-containing fraction (Fig. 3) was subjected to preparative HPLC over a Phenomenex Synergi-Max C_12_ preparative column using an isocratic system of 35:65 of CH_3_CN-H_2_O (0.1% formic acid) for 30 min at a flow rate of 21.2 ml min^−1^ to yield compounds **1** (60 mg), **2** (2.4 mg), **3** (6.0 mg), **4** (5.6 mg), **5** (5.2 mg), **6** (1.6 mg). The supernatant collected from the resuspension/centrifugation of the (5*Z*)-7-oxozeaenol-containing fraction (Fig. 4) was subjected to preparative HPLC over a Phenomenex Synergi-Max C_12_ preparative column using an isocratic system of 35:65 of CH_3_CN-H_2_O (0.1% formic acid) for 28 min, then to 80:20 of CH_3_CN-H_2_O (0.1% formic acid) over 5 min at a flow rate of 21.2 mL/min to yield compounds **1** (1.1 mg), **2** (137.9 mg), **7** (27.2 mg), **8** (10.0 mg), **9** (7.7 mg), **10** (2.1 mg), **11** (2.45 mg), **12** (1.9 mg), **13** (3.0 mg), **14** (1.4 mg), **15** (8.2 mg), **16** (4.0 mg), **17** (2.4 mg), **18** (2.0 mg), **19** (13.7 mg), **20** (1.9 mg).

### Palmariol C (19)

Compound **19** was isolated as a white amorphous powder, m.p. >260 °C with signs of decomposition; UV (CH_3_OH) *λ*_*max*_ (log *ε*) 205 (4.2), 257 (4.5), 291 (3.9), 302 (3.9), 342 (3.9) nm; IR v_max_ 3483, 2973, 2266, 1654, 1608, 1580, 1550, 1493, 1449, 1430, 1403 cm^−1^; ^1^H NMR (DMSO-*d*_6_, 400 MHz) and ^13^C NMR (DMSO-*d*_6_, 100 MHz) (see Table 1); HRESIMS *m/z* 307.0364 [M + H]^+^ (calcd. for C_15_H_12_ClO_5_, 307.0373).

### Palmariol D (20)

Compound **20** was isolated as a white amorphous powder, m.p. > 260 °C with signs of decomposition; UV (CH_3_OH) *λ*_*max*_ (log *ε*) 212 (4.0), 299 (3.7), 258 (4.0), 347 (4.0) nm; IR v_max_ 3300, 2924, 1650, 1598, 1582, 1546, 1509, 1463, 1425 cm^−1^; ^1^H NMR (DMSO-*d*_6_, 500 MHz) and ^13^C NMR (DMSO-*d*_6_, 125 MHz) (see Table 1); HRESIMS *m/z* 340.9974 [M + H]^+^ (calcd. for C_15_H_11_Cl_2_O_5_, 340.9984).

### Quantification of hypothemycin (1) and (5*Z*)-7-oxozeaenol (2)

Calibration curves of hypothemycin (**1**) and (5*Z*)-7-oxozeaenol (**2**) were developed using pure standards isolated from fungal strains MSX78495 and MSX63935, respectively. Ten standard solutions were prepared in CH_3_CN at a concentration range of 3–1536 ng ml^−1^. HRESIMS data were collected in triplicate *via* a UPLC-HRESIMS (Thermo LTQ Orbitrap XL) system using the area under the curve (AUC) to generate the calibration curves. A BEH Shield RP18 column (Waters, 1.7 µm; 50 × 2.1 mm) heated to 40 °C was utilized with a flow rate of the mobile phase of 0.3 ml min^−1^ and a gradient system of 15:85 to 100:0 of CH_3_CN-H_2_O (0.1% formic acid) over 10 min. MS data were collected from *m/z* 150 to 2000 in the positive mode. The linearity of each calibration curve, relative error (RE), and limit of quantitation (LOQ), which is the lowest amount of analyte that can be quantitatively determined with suitable precision and accuracy, were calculated and summarized (Fig. S7 and Table S3). Extracts from the fungal strains MSX78495, MSX63935, and MSX45109 were analyzed in triplicate, the areas were averaged, and the concentrations of **1** and **2** were extrapolated from the corresponding calibration curve. Statistical analysis was carried out using GraphPad Prism (GraphPad Software, La Jolla, CA), and comparisons were made using one-way ANOVA followed by Tukey post hoc test.

## Supplementary information

Supplemental Information

## Data Availability

Images of the three cis-enone RALs-producing fungi on different solid-state growth conditions, the extract amounts produced by these fungi, the UPLC chromatograms of culture extracts, calibration curves of hypothemycin and (5Z)-7-oxozeaenol, flash chromatography chromatogram of the extracts, H NMR spectra and UPLC purity of hypothemycin- and (5Z)-7-oxozeaenol-containing fractions, H NMR spectra of compounds **1**–**18**, and 1D and 2D NMR spectra of compounds **19** and **20**. The raw NMR spectra for **1**–**20** were deposited in Harvard Dataverse and can be freely accessed through 10.7910/DVN/XUQPLU.
