# Supplementary material for: Media and strain studies for the scaled production of cis-enone resorcylic acid lactones as feedstocks for semisynthesis
Source: J Antibiot (Tokyo). 2021 Jun 21;74(8):496–507. doi: 10.1038/s41429-021-00432-3 (PMC8313427; doi:10.1038/s41429-021-00432-3)
Supplement: Supplementary file 1 — Supplemental Information [file 41429_2021_432_MOESM1_ESM.docx]

**Supporting Information**

**Media and Strain Studies for the Scaled Production of *cis*-Enone Resorcylic Acid Lactones as Feedstocks for Semisynthesis**

Zeinab Y. Al Subeh,^a^ Huzefa A. Raja,^a^ Jennifer Obike,^a^ Cedric J. Pearce,^b^ and Mitchell P. Croatt,^a^ Nicholas H. Oberlies^*,a^

^a^Department of Chemistry and Biochemistry, The University of North Carolina at Greensboro, Greensboro, North Carolina 27402, United States.

^b^Mycosynthetix, Inc., Hillsborough, North Carolina 27278, United States.

^*^Corresponding author: Nicholas H. Oberlies ([nicholas_oberlies@uncg.edu](mailto:nicholas_oberlies@uncg.edu))

| **Supporting Information Contents** |
| --- |
| **Fig. S1.** HRESIMS data (base peak chromatograms) for the extracts of strains MSX78495, MSX63935, and MSX45109. |
| **Fig. S2.** The three *cis*-enone RAL-producing fungi grown in triplicates on three different media: rice, oatmeal, and Cheerios. |
| **Fig. S3.** Representative images of the three fungal strains MSX78495, MSX63935, and MSX45109 cultures grown on three different media. |
| **Fig. S4.** The extract amounts produced by the fungal strains MSX78495, MSX63935, and MSX45109 grown on rice, oatmeal, and Cheerios. |
| **Fig. S5.** UPLC-HRESIMS chromatograms of the extracts of the cultures from Fig. S4. |
| **Fig. S6.** The relative percentages of hypothemycin (**1**) and (5*Z*)-7-oxozeaenol (**2**), respectively, across cultures grown on rice, oatmeal, and Cheerios media. |
| **Table S1.** Analysis of the statistical difference of hypothemycin abundance among different cultures of fungal strains MSX78495, MSX63935, and MSX45109. |
| **Table S2.** Analysis of the statistical difference of (5*Z*)-7-oxozeaenol abundance among different cultures of strains MSX78495 and MSX63935. |
| **Fig. S7.** Calibration curves of hypothemycin and (5*Z*)-7-oxozeaenol developed to measure the absolute amounts for these two compounds across various extracts. |
| **Table S3.** The calibration curve parameters used for the quantitative analysis of hypothemycin (**1**) and (5*Z*)-7-oxozeaenol (**2**). |
| **Fig. S8.** First round of fractionation for the fungal extracts of MSX78495 via normal-phase flash chromatography to obtain six fractions. |
| **Fig. S9. A:** ^1^H NMR spectrum of fraction 4 from the flash chromatography shown in Fig. S7, demonstrating the purity of hypothemycin [400 MHz, CDCl_3_]. **B:** UPLC chromatogram (PDA detection) of fraction 4 from the flash chromatography shown in Fig. S7, demonstrating >97% purity. |
| **Fig. S10. A:** ^1^H NMR spectrum of fraction 3 from the flash chromatography shown in Fig. S7, demonstrating the purity of hypothemycin [400 MHz, CDCl_3_]. **B:** UPLC chromatogram (PDA detection) of fraction 3 from the flash chromatography shown in Fig. S7, demonstrating ~68% hypothemycin content. |
| **Fig. S11. A:** ^1^H NMR spectrum of the hypothemycin precipitate after two cycles of reconstitution and centrifugation in MeOH [400 MHz, CDCl_3_]. **B:** UPLC chromatograms (PDA detection) of the hypothemycin precipitate after two cycles of reconstitution and centrifugation in MeOH, demonstrating 94.4% pure hypothemycin sample. |
| **Fig. S12.** ^1^H NMR spectrum of the supernatants as compared to the precipitate after two cycles of reconstitution and centrifugation in MeOH [400 MHz, CDCl_3_]. |
| **Fig. S13.** Fractionation via flash chromatography for the fungal extract MSX63935 grown on rice. |
| **Fig. S14. A:** ^1^H NMR spectrum of fraction 3 from the flash chromatography shown in Fig. S12, demonstrating the purity of (5*Z*)-7-oxozeaenol [400 MHz, CDCl_3_]. **B:** UPLC chromatogram (PDA detection) of fraction 3 from the flash chromatography shown in Fig. S12, ~68% (5*Z*)-7-oxozeaenol content. |
| **Fig. S15. A:** ^1^H NMR spectrum of the (5*Z*)-7-oxozeaenol precipitate after four cycles of reconstitution and centrifugation in MeOH and CH_3_CN [400 MHz, CDCl_3_]. **B:** UPLC chromatograms (PDA detection) of the (5*Z*)-7-oxozeaenol precipitate after four cycles of reconstitution and centrifugation, demonstrating > 94% purity. |
| **Fig. S16.** ^1^H NMR spectrum of the MSX63935 supernatants collected after each cycle of the reconstitution and centrifugation process [400 MHz, CDCl_3_]. HPLC-grade MeOH was used in the first two cycles, while HPLC-grade CH_3_CN was used in the next two cycles. |
| **Fig. S17.** ^1^H and ^13^C NMR spectra of hypothemycin (**1**) [400 MHz for ^1^H and 100 MHz for ^13^C, CDCl_3_]. |
| **Fig. S18.** ^1^H and ^13^C NMR spectra of (5*Z*)-7-oxozeaenol (**2**) [400 MHz for ^1^H and 100 MHz for ^13^C, CDCl_3_]. |
| **Fig. S19.** ^1^H and ^13^C NMR spectra of dihydrohypothemycin (**3**) [400 MHz for ^1^H and 100 MHz for ^13^C, CDCl_3_]. |
| **Fig. S20.** ^1^H and ^13^C NMR spectra of aigialomycin A (**4**) [400 MHz for ^1^H and 100 MHz for ^13^C, CDCl_3_]. |
| **Fig. S21.** ^1^H and ^13^C NMR spectra of paecilomycin A (**5**) [400 MHz for ^1^H and 100 MHz for ^13^C, DMSO-*d*_6_]. |
| **Fig. S22.** ^1^H NMR spectrum of 4-O-demethylhypothemycin (**6**) [400 MHz, DMSO-*d*_6_]. |
| **Fig. S23.** ^1^H and ^13^C NMR spectra of (5*E*)-7-oxozeaenol (**7**) [400 MHz for ^1^H and 100 MHz for ^13^C, DMSO-*d*_6_]. |
| **Fig. S24.** ^1^H and ^13^C NMR spectra of LL-Z1640-1 (**8**) [500 MHz for ^1^H and 125 MHz for ^13^C, CDCl_3_]. |
| **Fig. S25.** ^1^H and ^13^C NMR spectra of zeaenol (**9**) [400 MHz for ^1^H and 100 MHz for ^13^C, CDCl_3_]. |
| **Fig. S26.** ^1^H and ^13^C NMR spectra of 7-*epi*-zeaenol (**10**) [400 MHz for ^1^H and 100 MHz for ^13^C, DMSO-*d*_6_]. |
| **Fig. S27.** ^1^H and ^13^C NMR spectra of aigialomycin B (**11**) [400 MHz for ^1^H and 100 MHz for ^13^C, CDCl_3_]. |
| **Fig. S28.** ^1^H and ^13^C NMR spectra of cochliomycin F (**12**) [400 MHz for ^1^H and 100 MHz for ^13^C, DMSO-*d*_6_]. |
| **Fig. S29.** ^1^H and ^13^C NMR spectra of radicinin (**13**) [500 MHz for ^1^H and 125 MHz for ^13^C, CDCl_3_]. |
| **Fig. S30.** ^1^H and ^13^C NMR spectra of dihydroradicinin (**14**) [400 MHz for ^1^H and 100 MHz for ^13^C, CDCl_3_]. |
| **Fig. S31.** ^1^H and ^13^C NMR spectra of alternariol (**15**) [500 MHz for ^1^H and 125 MHz for ^13^C, DMSO-*d*_6_]. |
| **Fig. S32.** ^1^H and ^13^C NMR spectra of alternariol 9-methyl ether (**16**) [500 MHz for ^1^H and 125 MHz for ^13^C, DMSO-*d*_6_]. |
| **Fig. S33.** ^1^H and ^13^C NMR spectra of rhizopycnin D (**17**) [500 MHz for ^1^H and 125 MHz for ^13^C, DMSO-*d*_6_]. |
| **Fig. S34.** ^1^H and ^13^C NMR spectra of palmariol B (**18**) [500 MHz for ^1^H and 125 MHz for ^13^C, DMSO-*d*_6_]. |
| **Fig. S35.** Mass spectrum of palmariol C (**19**). |
| **Fig. S36.** ^1^H and ^13^C NMR spectra of palmariol C (**19)** [400 MHz for ^1^H and 100 MHz for ^13^C, DMSO-*d*_6_]. |
| **Fig. S37.** Edited-HSQC NMR spectrum of palmariol C (**19)** [400 MHz, DMSO-*d*_6_]. |
| **Fig. S38.** COSY NMR spectrum of palmariol C (**19)** [400 MHz, DMSO-*d*_6_]. |
| **Fig. S39.** HMBC NMR spectrum of palmariol C (**19)** [400 MHz, DMSO-*d*_6_]. |
| **Fig. S40.** NOESY NMR spectrum of palmariol C (**19)** [400 MHz, DMSO-*d*_6_]. |
| **Fig. S41.** Mass spectrum of palmariol D (**20**). |
| **Fig. S42.**^1^H and ^13^C NMR spectra of palmariol D (**20)** [500 MHz for ^1^H and 125 MHz for ^13^C, DMSO-*d*_6_]. |
| **Fig. S43.** Edited-HSQC NMR spectrum of palmariol D (**20)** [400 MHz, DMSO-*d*_6_]. |
| **Fig. S44.** HMBC NMR spectrum of palmariol D (**20)** [400 MHz, DMSO-*d*_6_]. |
| **Fig. S45.** NOESY NMR spectrum of palmariol D (**20)** [400 MHz, DMSO-*d*_6_]. |
| **Fig. S46.** Key COSY and HMBC correlations of compounds **19** and **20**. |
| **Fig. S47.** NOESY correlations of compounds **19** and **20**. |
| **Fig. S48.** Analytical vs Prep HPLC chromatograms for a hypothemycin-containing fraction. |
| **Fig. S49.** Analytical vs Prep HPLC chromatograms for a (5*Z*)-7-oxozeaenol-containing fraction. |
| **Table S4.** Comparison between the application of reverse-phase HPLC vs. resuspension/ centrifugation techniques in the purification process of hypothemycin and (5*Z*)-7-oxozeaenol. |
| **Fig. S50.** Molecular phylogenetic analysis of fungal ITS sequences reveal MSX63935, MSX78495, and MSX45109 are members of the genus, *Setophoma* (*Phaeosphaeriaceae, Ascomycot*a). |

| **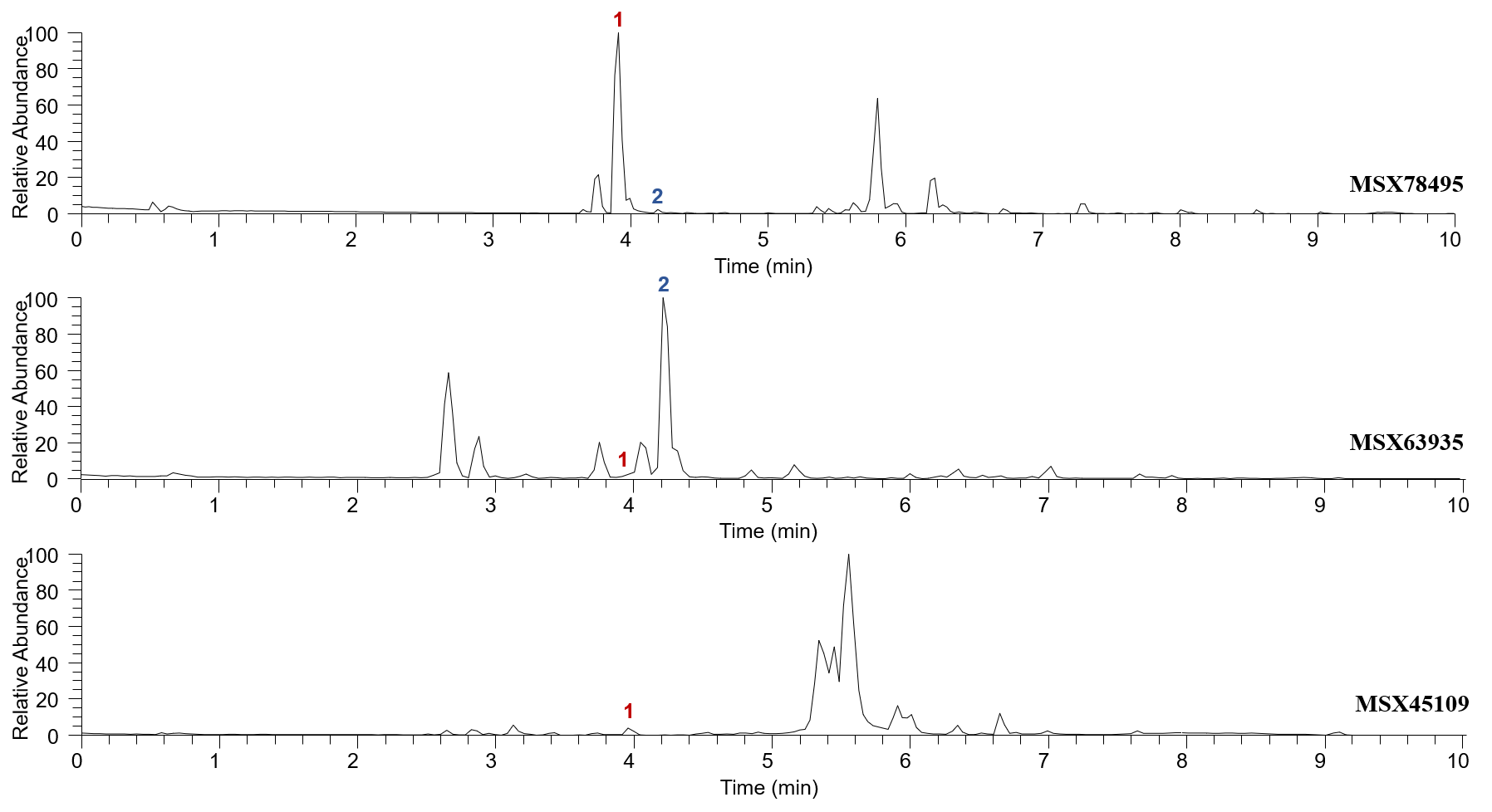** |
| --- |
| **Fig. S1.** HRESIMS data (base peak chromatograms) for the extracts of strains MSX78495, MSX63935, and MSX45109, where the presence of hypothemycin (**1**) and (5*Z*)-7-oxozeaenol (**2**) were identified via dereplication. |

| **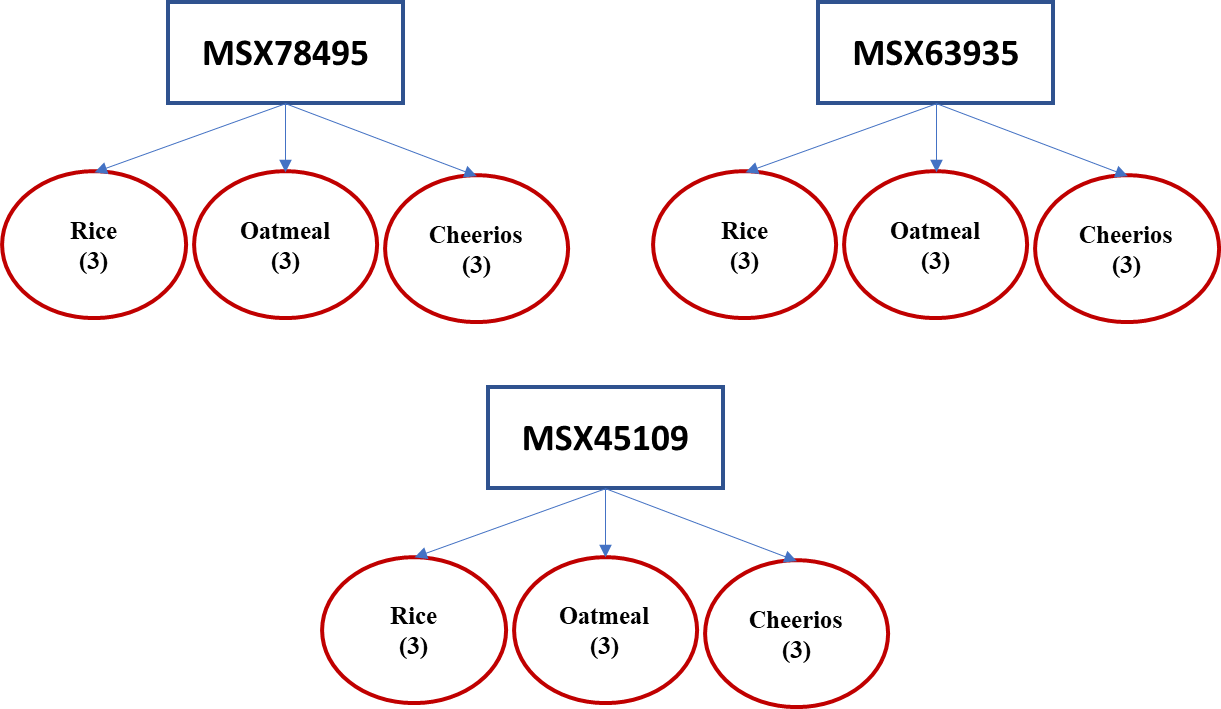** |
| --- |
| **Fig. S2.** The three *cis*-enone RALs-producing fungi grown in triplicates on three different media: rice, oatmeal, and Cheerios. |

| **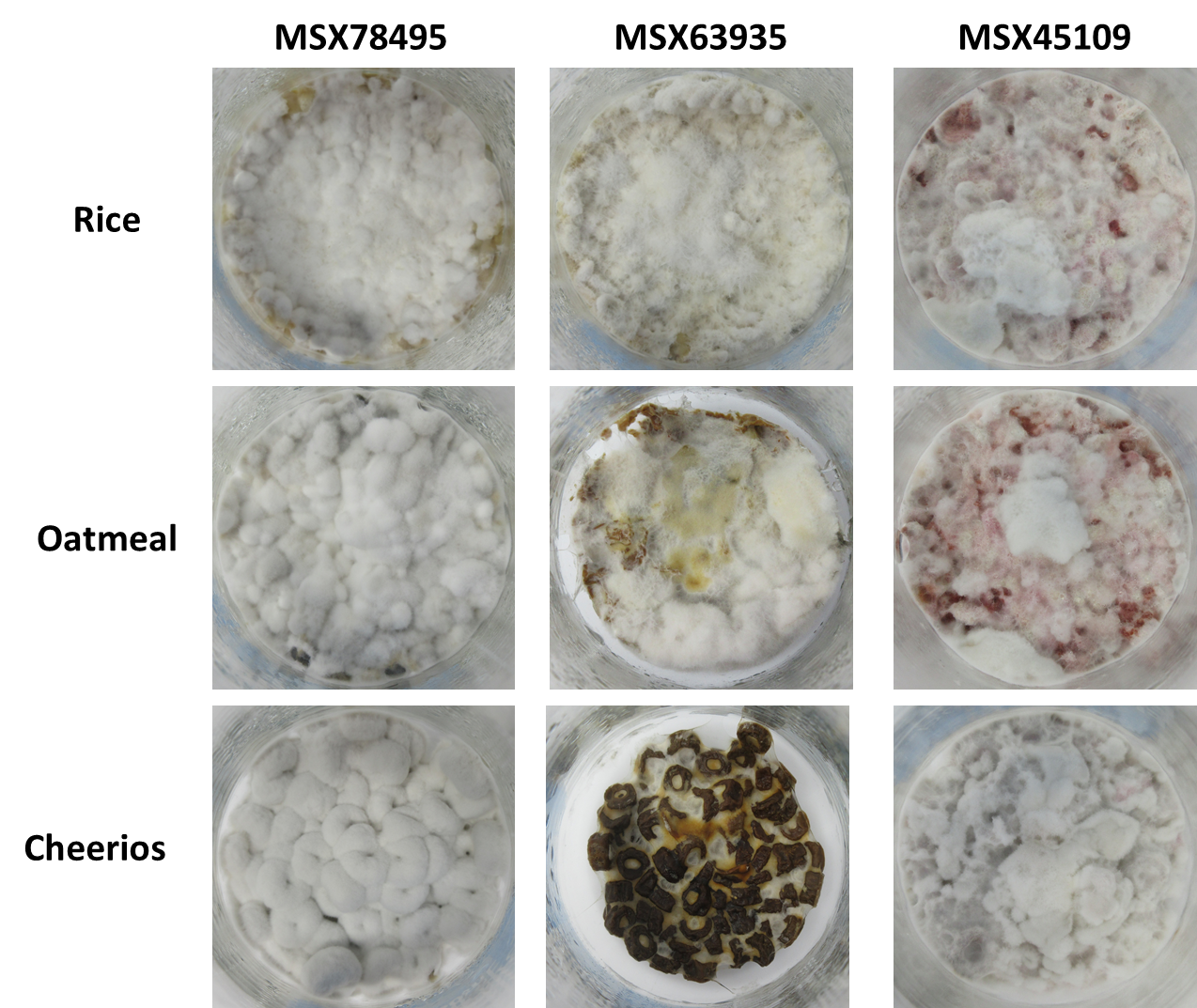** |
| --- |
| **Fig. S3.** Representative images of the three fungal strains MSX78495, MSX63935, and MSX45109 grown on three different media (i.e. rice, oatmeal, and Cheerios). These photographs were taken looking down the neck of the Erlenmeyer flasks after the samples had been growing for 14 days. |

| **** |
| --- |
| **Extract amount (mg) obtained from strains MSX78495, MSX63935, and MSX45109 grown on rice, oatmeal, and Cheerios.**  **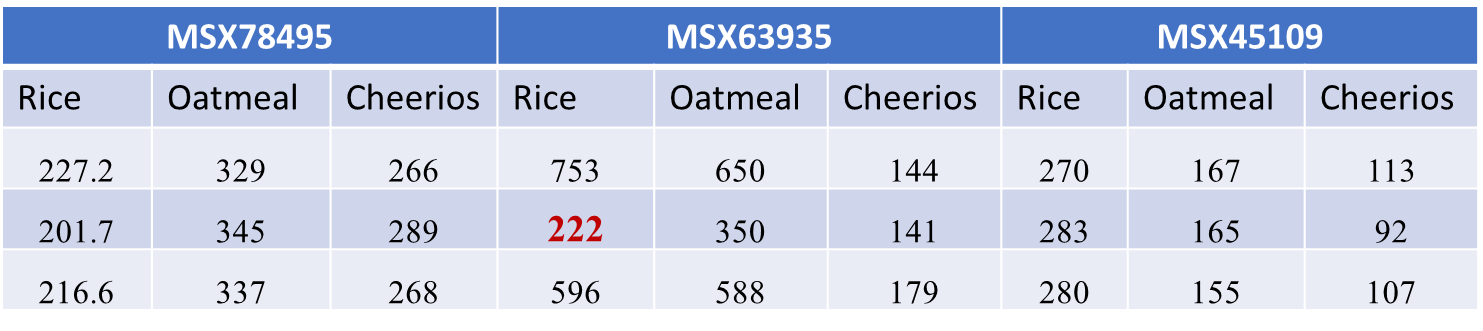** |
| **Fig. S4.** The extract amounts produced by the fungal strains MSX78495, MSX63935, and MSX45109 grown on rice, oatmeal, and Cheerios. The error bars are based on three biological replicates, each analyzed in triplicate. The value highlighted in red was considered an outlier and was responsible for the large error bars associated with strain MSX63935. That culture was not used in the purification studies for (5*Z*)-7-oxozeaenol. Data in the bar graph are presented as mean ± SD. |

| **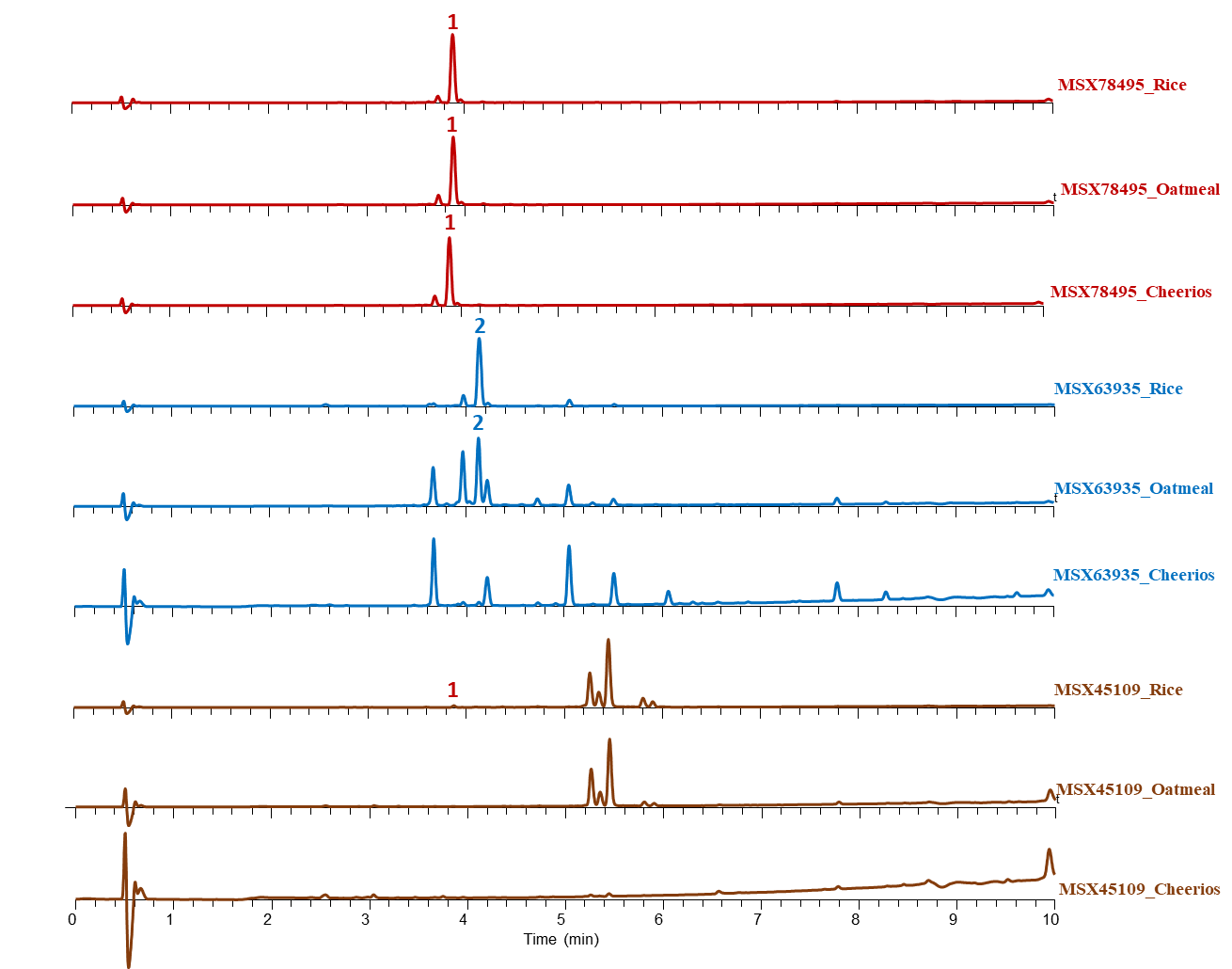** |
| --- |
| **Fig. S5.** UPLC-HRESIMS chromatograms of the extracts of the cultures from Fig. S4, all prepared at a concentration of 0.3 mg/mL. Each chromatogram represents an average of three biological replicates, and the annotation refers to hypothemycin (**1**) and (5*Z*)-7-oxozeaenol (**2**) as detected via dereplication.   \| **a** \|  \| **b** \|  \| \| --- \| --- \| --- \| --- \| \| **Fig. S6.** Panels a and b show the relative percentages of hypothemycin (**1**) and (5*Z*)-7-oxozeaenol (**2**), respectively, across cultures grown on rice, oatmeal, and Cheerios media. The relative percentages were measured by LC-HRESIMS in three biological replicates and multiplied by the extract weight, and then normalized according to the extract with highest abundance. * Indicates significantly higher productions of hypothemycin (**1**) by strain MSX78495 in panels A-B or (5*Z*)-7-oxozeaenol (**2**) by strain MSX63935 in panels C-D, compared to other fungal strains (*p* < 0.05) as demonstrated in table S1 and S2. Data are presented as mean ± SD. One of the three biological replicates of strain MSX63935 on rice medium did not show optimal growth, which caused the large error bars in panels b for the relative and absolute amount of **2** from strain MSX63935 grown on rice. \| \| \| \| |

| **Table S1.** Analysis of the statistical difference of hypothemycin abundance among different cultures of fungal strains MSX78495, MSX63935, and MSX45109. | | | | | | | | | |
| --- | --- | --- | --- | --- | --- | --- | --- | --- | --- |
| Culture type | Strain MSX78495 on rice | Strain MSX78495 on oatmeal | Strain MSX78495 on Cheerios | Strain MSX63935 on rice | Strain MSX63935 on oatmeal | Strain MSX63935 on Cheerios | Strain MSX45109 on rice | Strain MSX45109 on oatmeal | Strain MSX45109 on Cheerios |
| Strain MSX78495 on rice | -- | -- | -- | *p* < 0.05 | *p* < 0.05 | *p* < 0.05 | *p* < 0.05 | *p* < 0.05 | *p* < 0.05 |
| Strain MSX78495 on oatmeal | -- | -- | -- | *p* < 0.05 | *p* < 0.05 | *p* < 0.05 | *p* < 0.05 | *p* < 0.05 | *p* < 0.05 |
| Strain MSX78495 on Cheerios | -- | -- | -- | *p* < 0.05 | *p* < 0.05 | *p* < 0.05 | *p* < 0.05 | *p* < 0.05 | *p* < 0.05 |

| **Table S2.** Analysis of the statistical difference of (5*Z*)-7-oxozeaenol abundance among different cultures of strains MSX78495 and MSX63935. | | | | | | |
| --- | --- | --- | --- | --- | --- | --- |
| Culture type | Strain MSX78495 on rice | Strain MSX78495 on oatmeal | Strain MSX78495 on Cheerios | Strain MSX63935 on rice | Strain MSX63935 on oatmeal | Strain MSX63935 on Cheerios |
| Strain MSX63935 on rice | *p* < 0.05 | *p* < 0.05 | *p* < 0.05 | -- | -- | *p* < 0.05 |
| Strain MSX63935 on oatmeal | *p* < 0.05 | *p* < 0.05 | *p* < 0.05 | -- | -- | *p* < 0.05 |
| Strain MSX63935 on Cheerios | -- | -- | -- | *p* < 0.05 | *p* < 0.05 | -- |

| **A** | **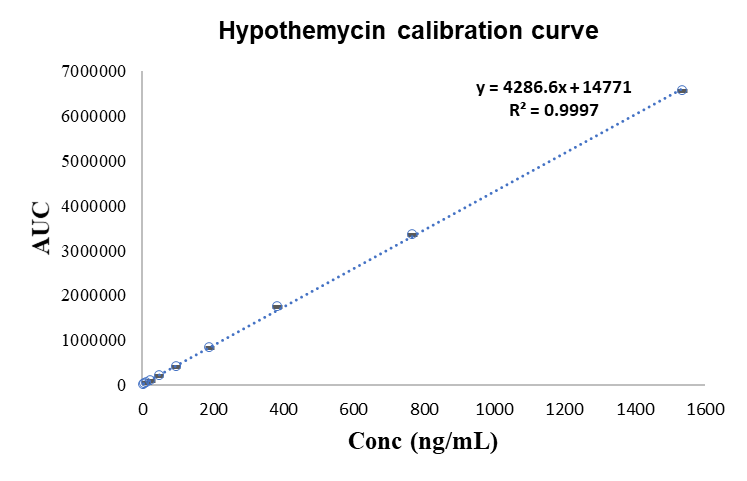** |
| --- | --- |
| **B** | **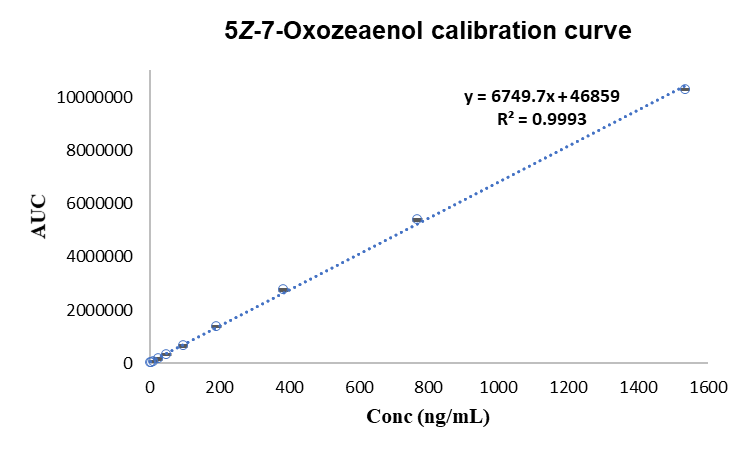** |
| **Fig. S7.** Calibration curves for hypothemycin (A) and (5*Z*)-7-oxozeaenol (B) developed to measure the absolute amounts for these two compounds across various extracts. Each standard was subjected thrice to UPLC-HRESIMS to measure the AUC. | |

| **Table S3.** The calibration curve parameters used for the quantitative analysis of hypothemycin (**1**) and (5*Z*)-7-oxozeaenol (**2**) | | |
| --- | --- | --- |
|  | **1** | **2** |
| Retention time (min) | 3.89 | 4.14 |
| Observed mass (*m/z*) | 379.13773 | 363.14285 |
| Calculated mass (*m/z*) | 379.1393 | 363.1443 |
| Linear equation | y = 4286.6x + 14771 | y = 6749.7x + 46859 |
| R^2^ | 0.9997 | 0.9993 |
| Range (ng/mL) | 3-1536 | 3-1536 |
| Relative error (RE) | <15% | <15% |
| Linearity range (ng/mL) | 48-1536 | 48-1536 |

| 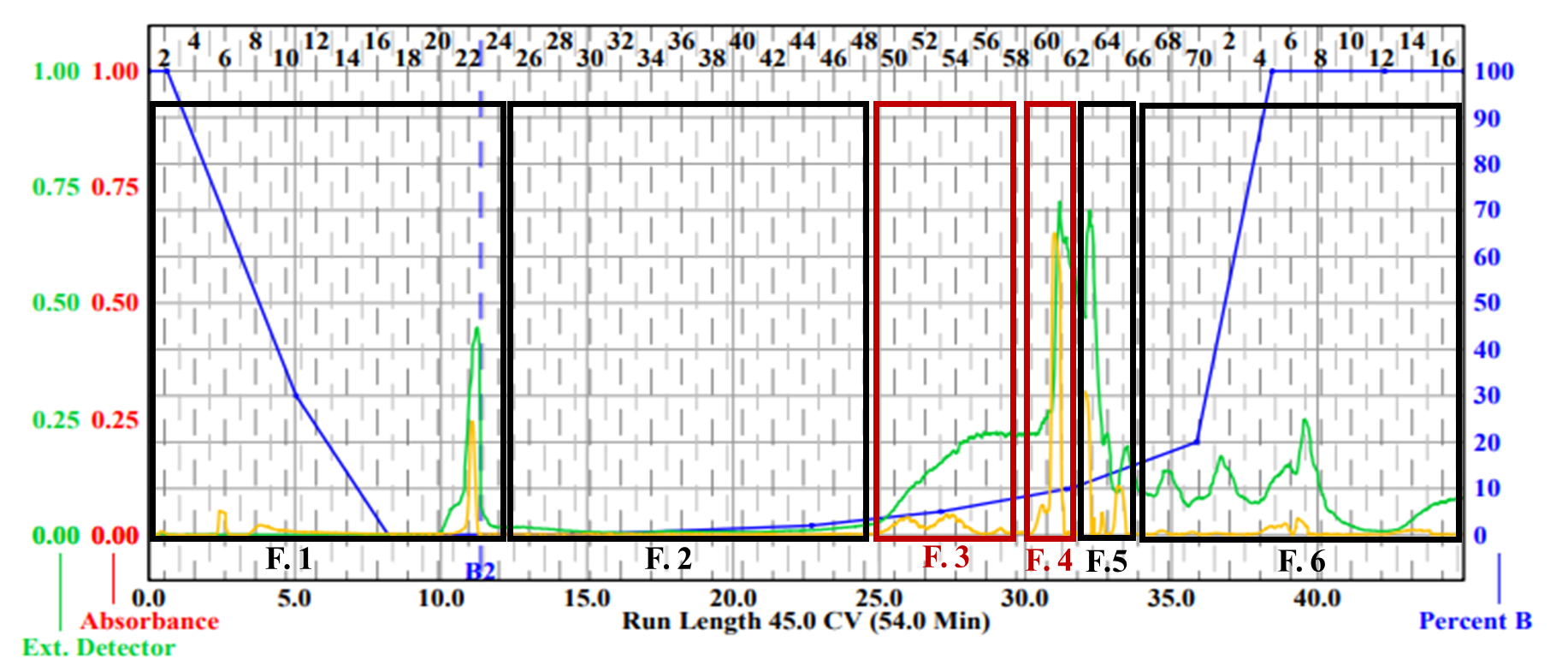 |
| --- |
| **Fig. S8.** First round of fractionation for the fungal extracts of MSX78495 via normal-phase flash chromatography to obtain six fractions (F.1-F.6). A gradient solvent system of hexanes-CHCl_3_-CH_3_OH at a 35 mL/min flow rate was used (blue line). The elution of compounds was monitored via ELSD detector (green line) and PDA detector (yellow line). |

| **A** |  |
| --- | --- |
| **B** | 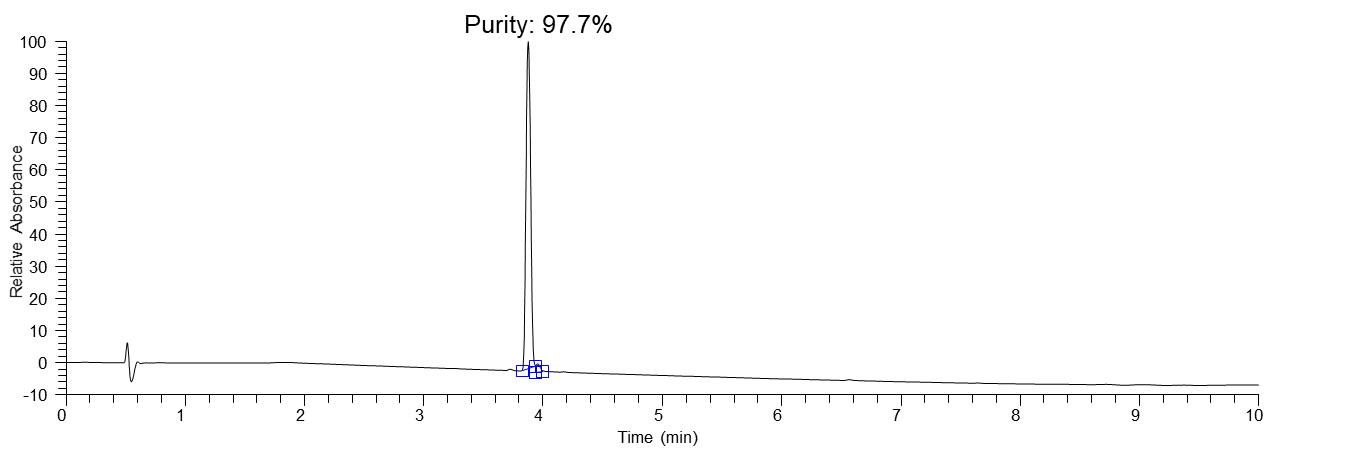 |
| **Fig. S9. A:** ^1^H NMR spectrum of fraction 4 from the flash chromatography shown in Fig. S7, demonstrating the purity of hypothemycin [400 MHz, CDCl_3_]. **B:** UPLC chromatogram (PDA detection) of fraction 4 from the flash chromatography shown in Fig. S7, demonstrating >97% purity. | |

| **A** |  |
| --- | --- |
| **B** | 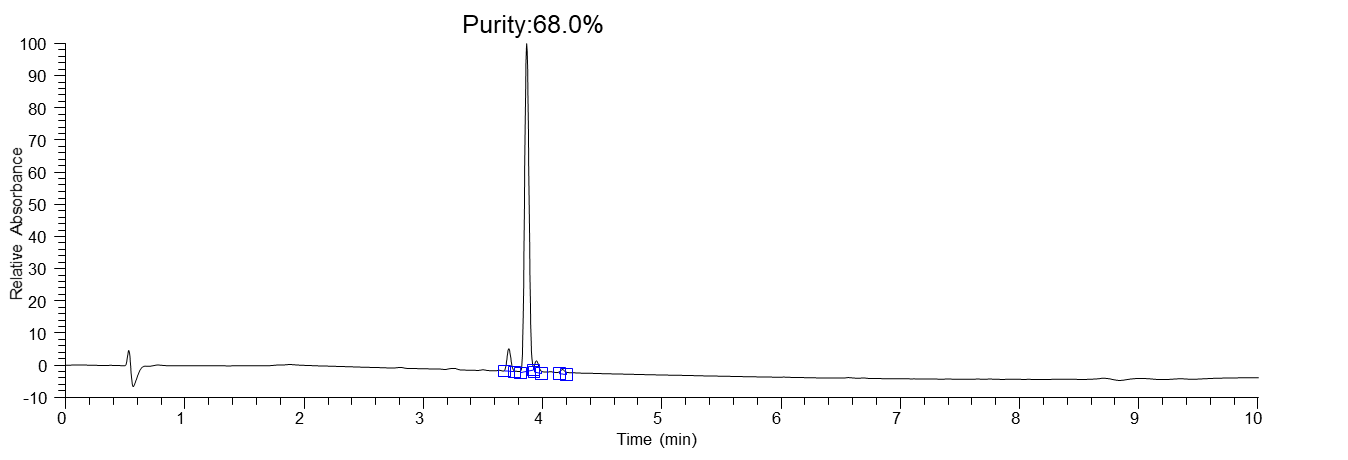 |
| **Fig. S10. A:** ^1^H NMR spectrum of fraction 3 from the flash chromatography shown in Fig. S7, demonstrating the purity of hypothemycin [400 MHz, CDCl_3_]. **B:** UPLC chromatogram (PDA detection) of fraction 3 from the flash chromatography shown in Fig. S7, demonstrating ~68% hypothemycin content. | |

| **A** |  |
| --- | --- |
| **B** | 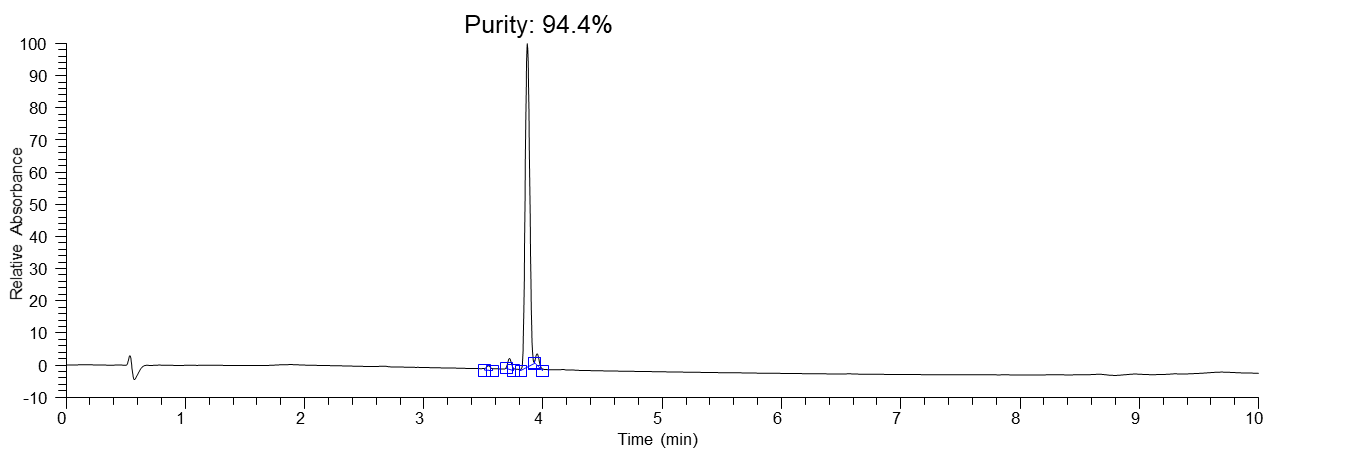 |
| **Fig. S11. A:** ^1^H NMR spectrum of the hypothemycin precipitate after two cycles of reconstitution and centrifugation in MeOH [400 MHz, CDCl_3_]. **B:** UPLC chromatograms (PDA detection) of the hypothemycin precipitate after two cycles of reconstitution and centrifugation in MeOH, demonstrating > 94% purity. | |

| 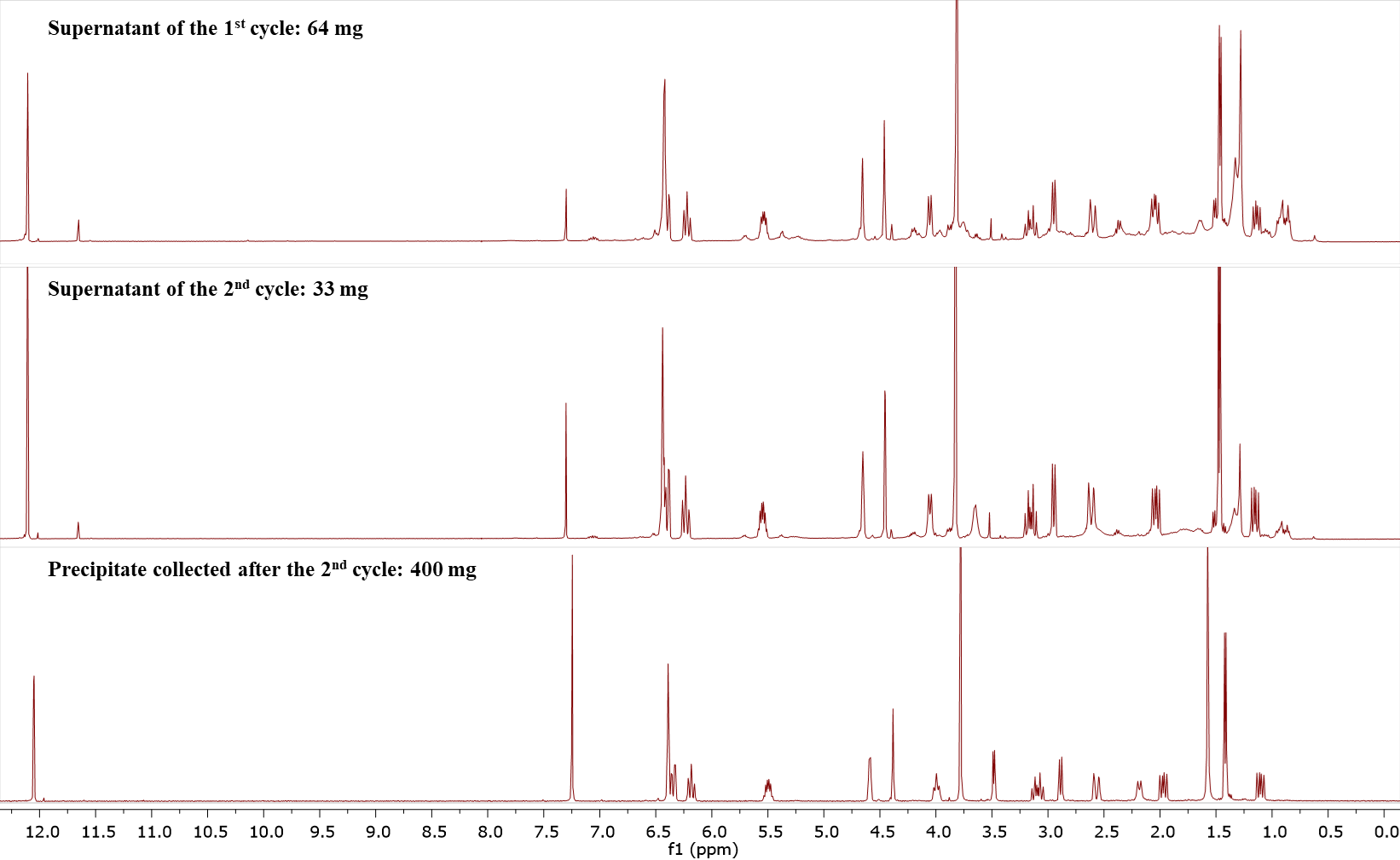 |
| --- |
| **Fig. S12.** ^1^H NMR spectrum of the supernatants as compared to the precipitate after two cycles of reconstitution and centrifugation in MeOH [400 MHz, CDCl_3_]. |

| 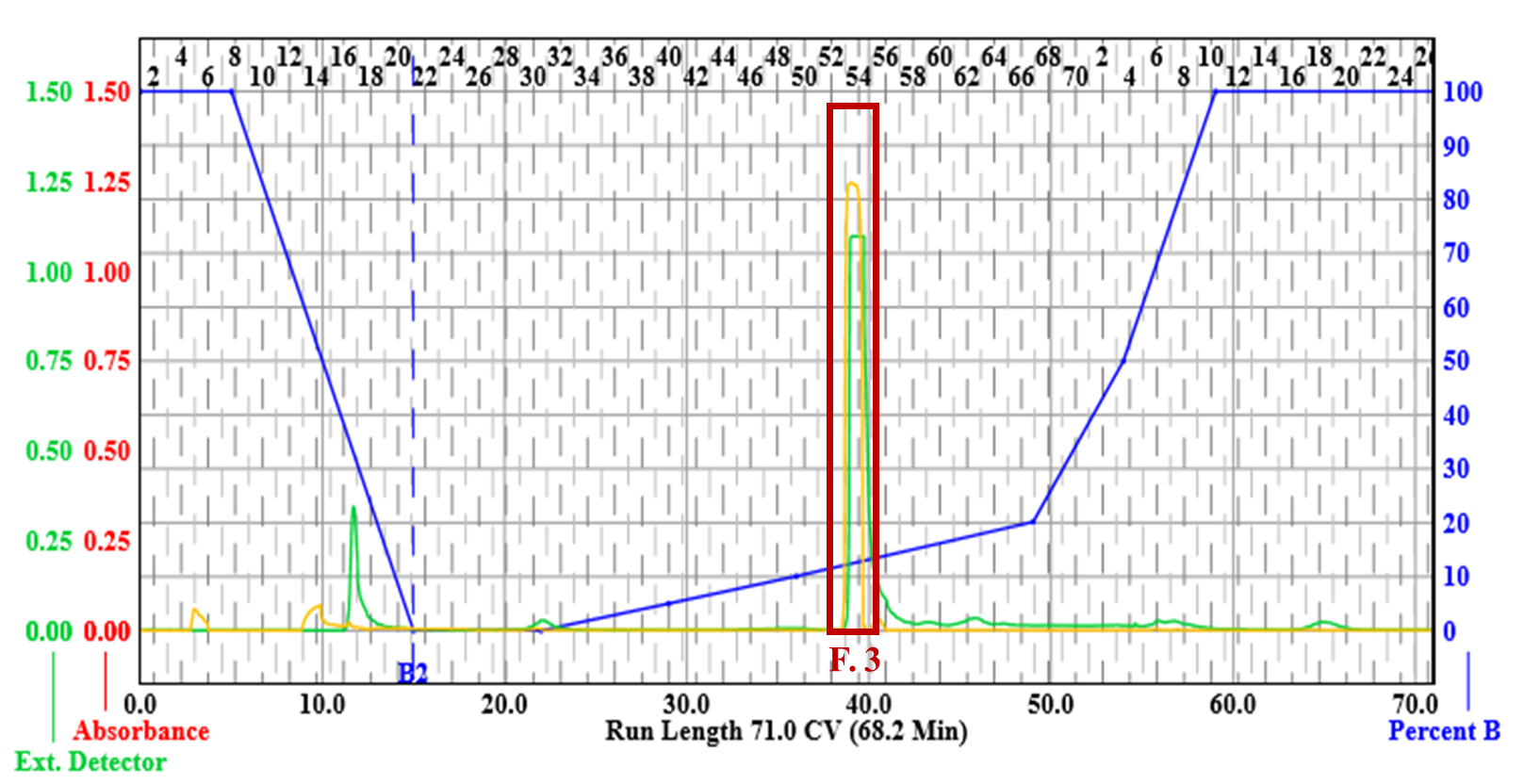 |
| --- |
| **Fig. S13.** Fractionation via normal-phase flash chromatography for the fungal extract MSX63935 grown on rice. A gradient solvent system of hexanes-CHCl_3_-CH_3_OH at a 35 mL/min flow rate was used (blue line). The elution of compounds was monitored via ELSD detector (green line) and PDA detector (yellow line). |

| **A** |  |
| --- | --- |
| **B** | 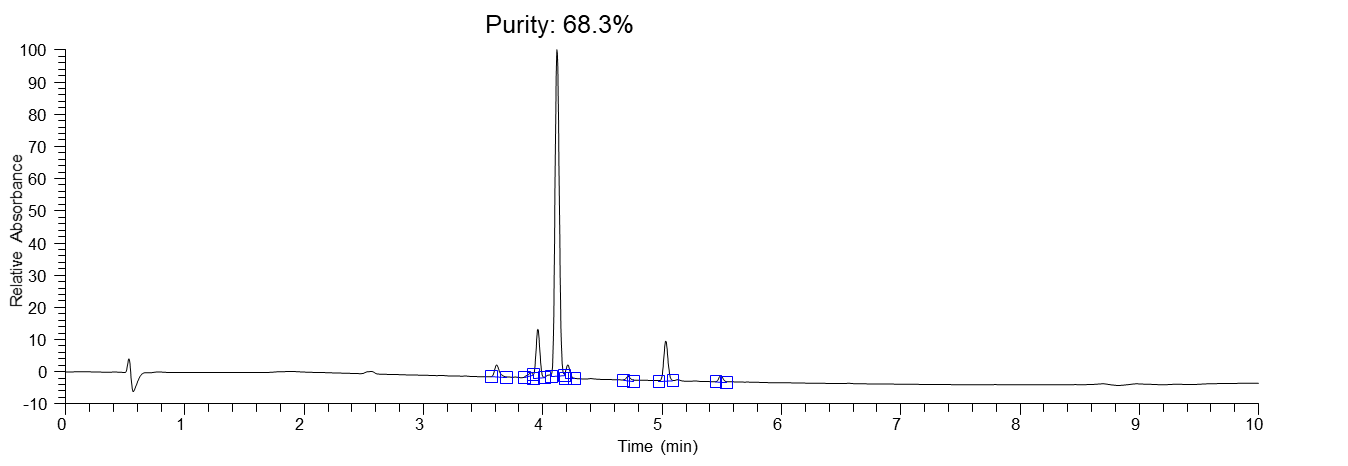 |
| **Fig. S14. A:** ^1^H NMR spectrum of fraction 3 from the flash chromatography shown in Fig. S12, demonstrating the purity of (5*Z*)-7-oxozeaenol [400 MHz, CDCl_3_]. **B:** UPLC chromatogram (PDA detection) of fraction 3 from the flash chromatography shown in Fig. S12, ~68% (5*Z*)-7-oxozeaenol content. | |

| **A** |  |
| --- | --- |
| **B** | 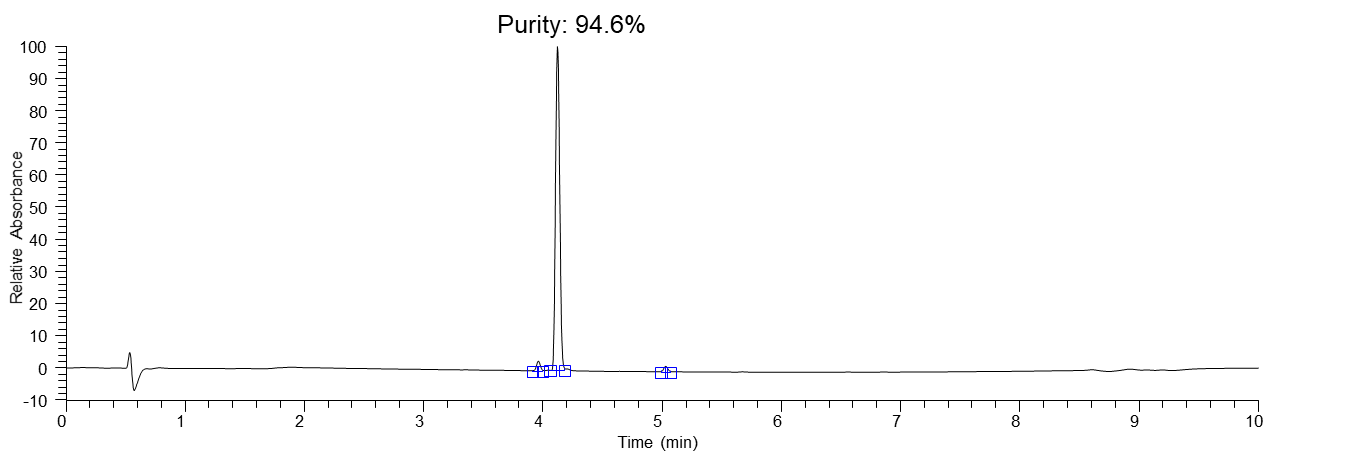 |
| **Fig. S15. A:** ^1^H NMR spectrum of the (5*Z*)-7-oxozeaenol precipitate after four cycles of reconstitution and centrifugation in MeOH and CH_3_CN [400 MHz, CDCl_3_]. **B:** UPLC chromatograms (PDA detection) of the (5*Z*)-7-oxozeaenol precipitate after four cycles of reconstitution and centrifugation, demonstrating > 94% purity. | |

| 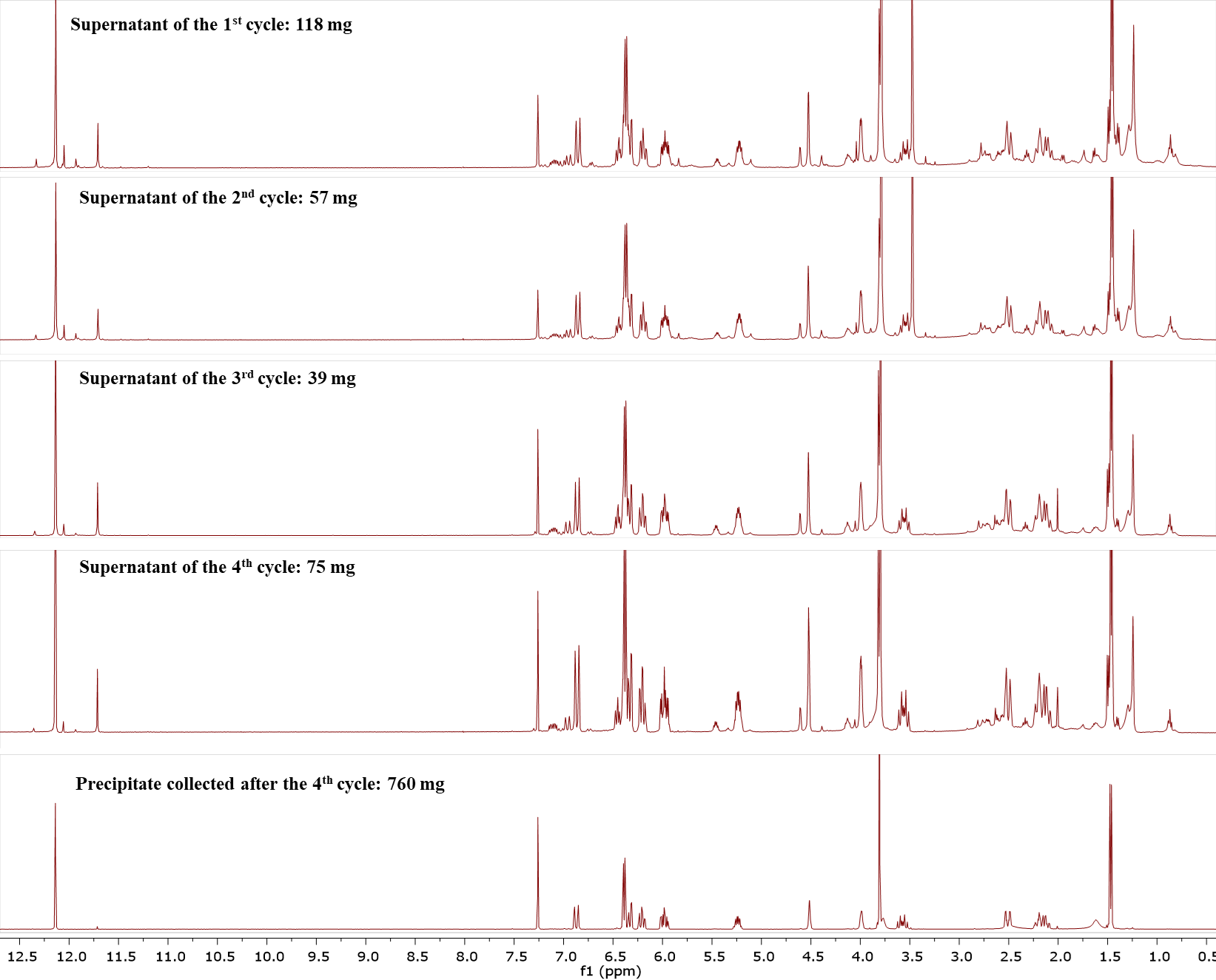 |
| --- |
| **Fig. S16.** ^1^H NMR spectrum of the MSX63935 supernatants collected after each cycle of the reconstitution and centrifugation process [400 MHz, CDCl_3_]. HPLC-grade MeOH was used in the first two cycles, while HPLC-grade CH_3_CN was used in the next two cycles. |

**Fig. S17.** ^1^H and ^13^C NMR spectra of hypothemycin (**1**) [400 MHz for ^1^H and 100 MHz for ^13^C, CDCl_3_].

**Fig. S18.** ^1^H and ^13^C NMR spectra of (5*Z*)-7-oxozeaenol (**2**) [400 MHz for ^1^H and 100 MHz for ^13^C, CDCl_3_].

**Fig. S19.** ^1^H and ^13^C NMR spectra of dihydrohypothemycin (**3**) [400 MHz for ^1^H and 100 MHz for ^13^C, CDCl_3_].


**Fig. S20.** ^1^H and ^13^C NMR spectra of aigialomycin A (**4**) [400 MHz for ^1^H and 100 MHz for ^13^C, CDCl_3_].

**Fig. S21.** ^1^H and ^13^C NMR spectra of paecilomycin A (**5**) [400 MHz for ^1^H and 100 MHz for ^13^C, DMSO-*d*_6_].

**Fig. S22.** ^1^H NMR spectrum of 4-O-demethylhypothemycin (**6**) [400 MHz, DMSO-*d*_6_].

**Fig. S23.** ^1^H and ^13^C NMR spectra of (5*E*)-7-oxozeaenol (**7**) [400 MHz for ^1^H and 100 MHz for ^13^C, DMSO-*d*_6_].

**Fig. S24.** ^1^H and ^13^C NMR spectra of LL-Z1640-1 (**8**) [500 MHz for ^1^H and 125 MHz for ^13^C, CDCl_3_].

**Fig. S25.** ^1^H and ^13^C NMR spectra of zeaenol (**9**) [400 MHz for ^1^H and 100 MHz for ^13^C, CDCl_3_].

**Fig. S26.** ^1^H and ^13^C NMR spectra of 7-*epi*-zeaenol (**10**) [400 MHz for ^1^H and 100 MHz for ^13^C, DMSO-*d*_6_].

**Fig. S27.** ^1^H and ^13^C NMR spectra of aigialomycin B (**11**) [400 MHz for ^1^H and 100 MHz for ^13^C, CDCl_3_].

**Fig. S28.** ^1^H and ^13^C NMR spectra of cochliomycin F (**12**) [400 MHz for ^1^H and 100 MHz for ^13^C, DMSO-*d*_6_].

**Fig. S29.** ^1^H and ^13^C NMR spectra of radicinin (**13**) [500 MHz for ^1^H and 125 MHz for ^13^C, CDCl_3_].

**Fig. S30.** ^1^H and ^13^C NMR spectra of dihydroradicinin (**14**) [400 MHz for ^1^H and 100 MHz for ^13^C, CDCl_3_].

**Fig. S31.** ^1^H and ^13^C NMR spectra of alternariol (**15**) [500 MHz for ^1^H and 125 MHz for ^13^C, DMSO-*d*_6_].

**Fig. S32.** ^1^H and ^13^C NMR spectra of alternariol 9-methyl ether (**16**) [500 MHz for ^1^H and 125 MHz for ^13^C, DMSO-*d*_6_].

**Fig. S33.** ^1^H and ^13^C NMR spectra of rhizopycnin D (**17**) [500 MHz for ^1^H and 125 MHz for ^13^C, DMSO-*d*_6_].

**Fig. S34.** ^1^H and ^13^C NMR spectra of palmariol B (**18**) [500 MHz for ^1^H and 125 MHz for ^13^C, DMSO-*d*_6_].

| 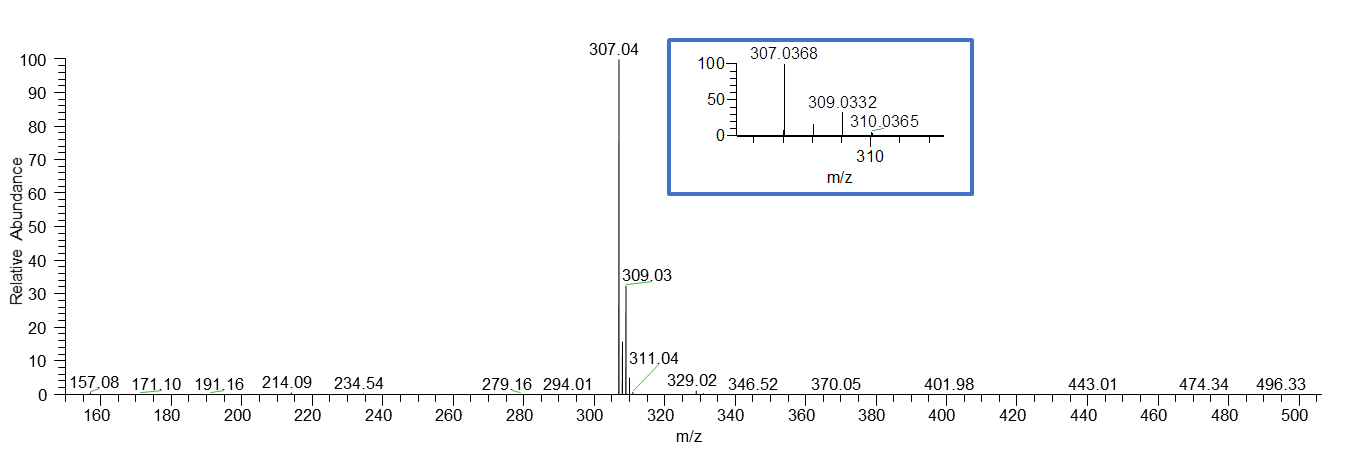 |
| --- |
| **Fig. S35.** Mass spectrum of palmariol C (**19**). |

**Fig. S36.**^1^H and ^13^C NMR spectra of palmariol C (**19)** [400 MHz for ^1^H and 100 MHz for ^13^C, DMSO-*d*_6_].

**Fig. S37.** Edited-HSQC NMR spectrum of palmariol C (**19)** [400 MHz, DMSO-*d*_6_].

**Fig. S38.** COSY NMR spectrum of palmariol C (**19)** [400 MHz, DMSO-*d*_6_].

**Fig. S39.** HMBC NMR spectrum of palmariol C (**19)** [400 MHz, DMSO-*d*_6_].

**Fig. S40.** NOESY NMR spectrum of palmariol C (**19)** [400 MHz, DMSO-*d*_6_].

| 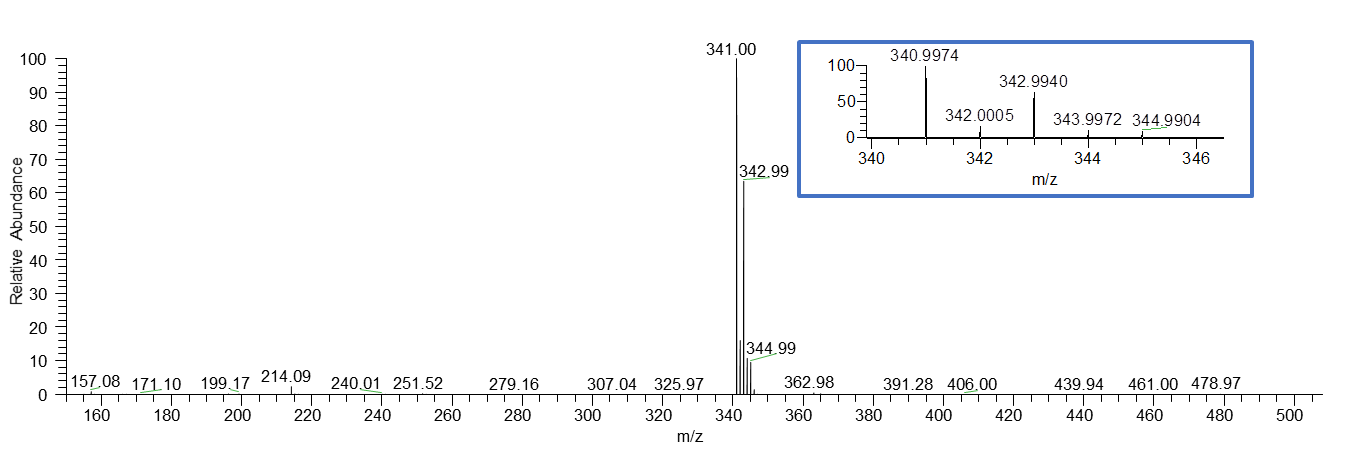 |
| --- |
| **Fig. S41.** Mass spectrum of palmariol D (**20**). |

**Fig. S42.**^1^H and ^13^C NMR spectra of palmariol D (**20)** [500 MHz for ^1^H and 125 MHz for ^13^C, DMSO-*d*_6_].

**Fig. S43.** Edited-HSQC NMR spectrum of palmariol D (**20)** [400 MHz, DMSO-d_6_].

**Fig. S44.** HMBC NMR spectrum of palmariol D (**20)** [400 MHz, DMSO-*d*_6_].

**Fig. S45.** NOESY NMR spectrum of palmariol D (**20)** [400 MHz, DMSO-*d*_6_].

|  |  |
| --- | --- |
| **19** | **20** |
| **Fig. S46.** Key COSY and HMBC correlations of compounds **19** and **20**. | |
|  |  |
| **19** | **20** |
| **Fig. S47.** NOESY correlations of compounds **19** and **20**. | |

| 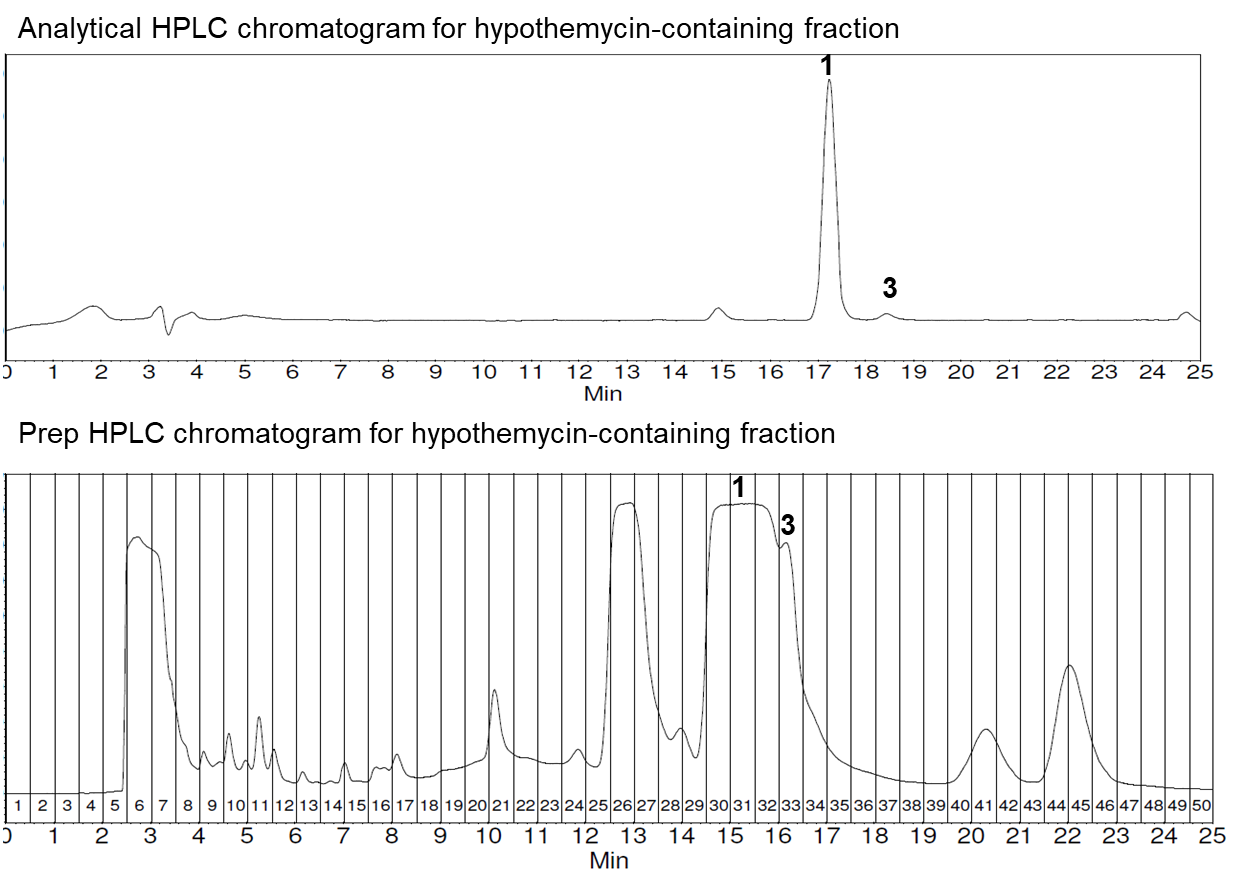 |
| --- |
| **Fig. S48.** Analytical vs Prep HPLC chromatograms for hypothemycin-containing fraction demonstrating a co-elution of dihydrohypothemycin (**3**) with hypothemycin (**1**) despite the optimization of the analytical method. |

| 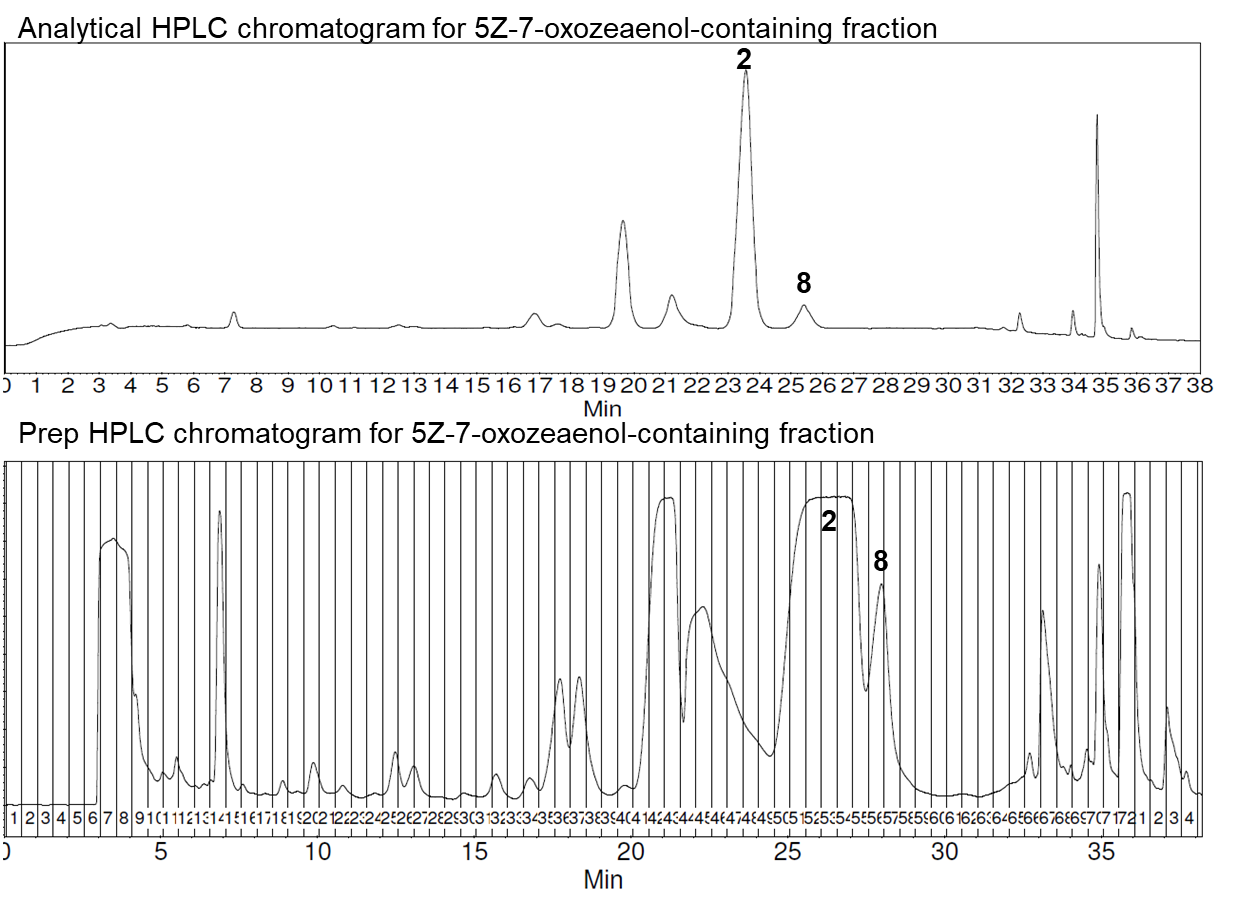 |
| --- |
| **Fig. S49.** Analytical vs Prep HPLC chromatograms for a (5*Z*)-7-oxozeaenol-containing fraction demonstrating a co-elution of LL-Z1640-1 (**8**) with (5*Z*)-7-oxozeaenol (**2**) despite the optimization of the analytical method. |

| **Table S4.** Comparison between the application of reverse-phase HPLC vs. resuspension/ centrifugation techniques in the purification process of hypothemycin and (5*Z*)-7-oxozeaenol | | | | |
| --- | --- | --- | --- | --- |
| **parameters** | **Reverse-phase HPLC** | | **Resuspension/centrifugation approach** | |
|  | Hypothemycin | (5*Z*)-7-oxozeaenol | Hypothemycin | (5*Z*)-7-oxozeaenol |
| Number of injections | 10-15 | 10-15 | -- | -- |
| Amount per injection (mg) | 50-100 mg | 50-100 mg | -- | -- |
| Estimated usage of HPLC- grade acetonitrile | 2.5-3.5 L | 3.5-5.0 L | -- | 12 mL |
| Estimated usage of DI H_2_O (0.1% formic acid) | 4.1-6.2 L | 6.2-9.3 L | -- | -- |
| Estimated usage of HPLC-grade methanol | -- | -- | 10 mL | -- |
| Average extraction-to-purification time | 2-3 weeks | 2-3 weeks | 3-4 days | 3-4 days |
| Instrumentation time on the HPLC | 7-10 hr | 9-14 hr | -- | -- |
| Instrumentation time on the rotavap | 2-3 days | 2-3 days | -- | -- |

| 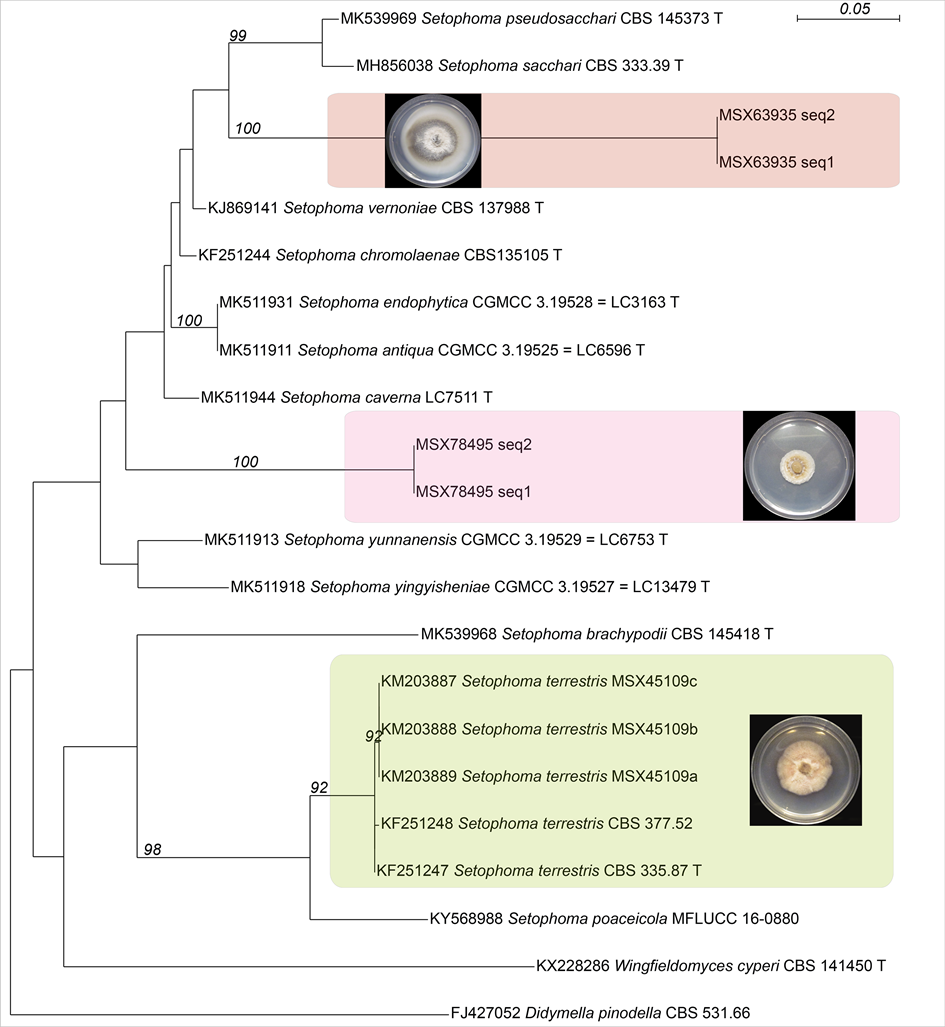 |
| --- |
| **Fig. S50.** Molecular phylogenetic analysis of fungal ITS sequences reveal MSX63935, MSX78495, and MSX45109 are members of the genus, *Setophoma* (*Phaeosphaeriaceae, Ascomycot*a). Phylogram of the most likely tree (−lnL = 2720.466) from a Maximum Likelihood analysis of 22 sequences based on the ITS region (505 bp) using IQ-TREE. Numbers refer to UFBoot support values ≥ 90% based on 5000 replicates. Nodes ≥95 are considered strongly supported. Three week old, malt extract agar cultures of MSX63935 and MSX78495 and potato dextrose agar culture of MSX45109 are shown. *Didymella pinodella* CBS 531.66 was used as outgroup. Ex-type isolates are designated by the letter T. Bar indicates nucleotide substitutions per site. |
